# An optimized transformation protocol for *Anthoceros agrestis* and three more hornwort species

**DOI:** 10.1111/tpj.16161

**Published:** 2023-04-11

**Authors:** Manuel Waller, Eftychios Frangedakis, Alan O. Marron, Susanna Sauret‐Güeto, Jenna Rever, Cyrus Raja Rubenstein Sabbagh, Julian M. Hibberd, Jim Haseloff, Karen S. Renzaglia, Péter Szövényi

**Affiliations:** ^1^ Department of Systematic and Evolutionary Botany University of Zurich Zurich Switzerland; ^2^ Zurich‐Basel Plant Science Center Zurich Switzerland; ^3^ Department of Plant Sciences University of Cambridge Downing Street Cambridge CB2 3EA UK; ^4^ Department of Microbiology and Molecular Genetics, College of Biological Sciences University of California Davis California 95616 USA; ^5^ Department of Plant Biology Southern Illinois University Carbondale Illinois 62901 USA; ^6^ Present address: Crop Science Centre University of Cambridge 93 Lawrence Weaver Road Cambridge CB3 0LE UK

**Keywords:** *Agrobacterium*‐mediated transformation, hornworts, subcellular targeting, constitutive promoter, land plant evolution

## Abstract

Land plants comprise two large monophyletic lineages, the vascular plants and the bryophytes, which diverged from their most recent common ancestor approximately 480 million years ago. Of the three lineages of bryophytes, only the mosses and the liverworts are systematically investigated, while the hornworts are understudied. Despite their importance for understanding fundamental questions of land plant evolution, they only recently became amenable to experimental investigation, with *Anthoceros agrestis* being developed as a hornwort model system. Availability of a high‐quality genome assembly and a recently developed genetic transformation technique makes *A. agrestis* an attractive model species for hornworts. Here we describe an updated and optimized transformation protocol for *A. agrestis*, which can be successfully used to genetically modify one more strain of *A. agrestis* and three more hornwort species, *Anthoceros punctatus*, *Leiosporoceros dussii*, and *Phaeoceros carolinianus.* The new transformation method is less laborious, faster, and results in the generation of greatly increased numbers of transformants compared with the previous method. We have also developed a new selection marker for transformation. Finally, we report the development of a set of different cellular localization signal peptides for hornworts providing new tools to better understand the hornwort cell biology.

## Significance Statement

Hornworts, despite their key phylogenetic position and their unique biology, have been widely overlooked by the plant biology community. Here we report a greatly optimized transformation technique applicable for several hornwort species (*Anthoceros agrestis*, *Anthoceros punctatus*, *Leiosporoceros dussi*, and *Phaeoceros carolinianus*) as well as provide subcellular localization signals, a new selection marker, and additional constitutive promoters to be used.

## INTRODUCTION

Hornworts, together with the mosses and liverworts, belong to bryophytes, a monophyletic group sister to all other land plants (tracheophytes). Bryophytes are crucial for revealing the nature of the land plant common ancestor and improving our understanding of fundamental land plant evolutionary innovations (Puttick et al., 2018). There is mounting evidence that morphological, and developmental characters were independently lost and gained in each of the three lineages of bryophytes (Clark et al., 2022; Harris et al., 2022; Li et al., 2020). Such an example is stomata that are present in hornworts and mosses but are absent in liverworts (Frangedakis, Shimamura, et al., 2021; Harrison & Morris, 2018; Ligrone et al., 2012). Therefore, studying representative species from each of the three main bryophyte clades is necessary to understand the evolution and the likely ancestral state of a trait of interest. Hornworts can be instrumental in understanding the evolution of: (i) sporophyte; (ii) plant–microbe symbiosis; and (iii) plastids. Sporophytes of hornworts are morphologically and developmentally distinct from other bryophytes and land plants. In particular, in hornworts, the sporophyte grows from a multicellular basal meristem, whereas in mosses from an intercalary meristem (Frangedakis, Shimamura, et al., 2021; Harrison & Morris, 2018; Ligrone et al., 2012), and in the liverwort *Marchantia polymorpha* a distinct sporophytic multicellular proliferative region is absent. In contrast, in vascular plants the sporophyte grows from a shoot and root apical meristem. Hornworts establish symbiotic interactions with mycorrhizal fungi (Desiro et al., 2013; Frangedakis, Shimamura, et al., 2021; Read et al., 2000) but are also able to form symbiotic interactions with cyanobacteria, which occur only in a few, phylogenetically separated lineages of land plants (Adams, 2002; Chatterjee et al., 2022; Duggan et al., 2013). Finally, hornworts are unique among land plants as they have a single chloroplast per cell that contains a pyrenoid based alga‐like carbon‐concentrating mechanism (Li et al., 2017; Vaughn et al., 1992). Understanding the genetic pathways controlling the development of these unique traits in hornworts, and comparing them to mosses, liverworts as well as vascular plants, can greatly facilitate efforts towards a more comprehensive understanding of the mechanisms underpinning land plant evolution.

However, until recently, a tractable model system was lacking for hornworts. To further our understanding of hornwort biology, *Anthoceros agrestis* was recently proposed as an experimental model system (Frangedakis, Shimamura, et al., 2021; Gunadi et al., 2022; Li et al., 2020; Neubauer et al., 2022; Szövényi et al., 2015) with two geographic isolates (Oxford and Bonn) available. Axenic culturing methods for both isolates have been established and their genomes have been sequenced. Importantly, an *Agrobacterium*‐mediated stable transformation method has been developed (Frangedakis, Waller, et al., 2021). Transgenic lines can be propagated vegetatively without losing the transgene. These technical advances provide us with the tools to unlock fundamental questions of hornwort biology.

**Figure 1:**
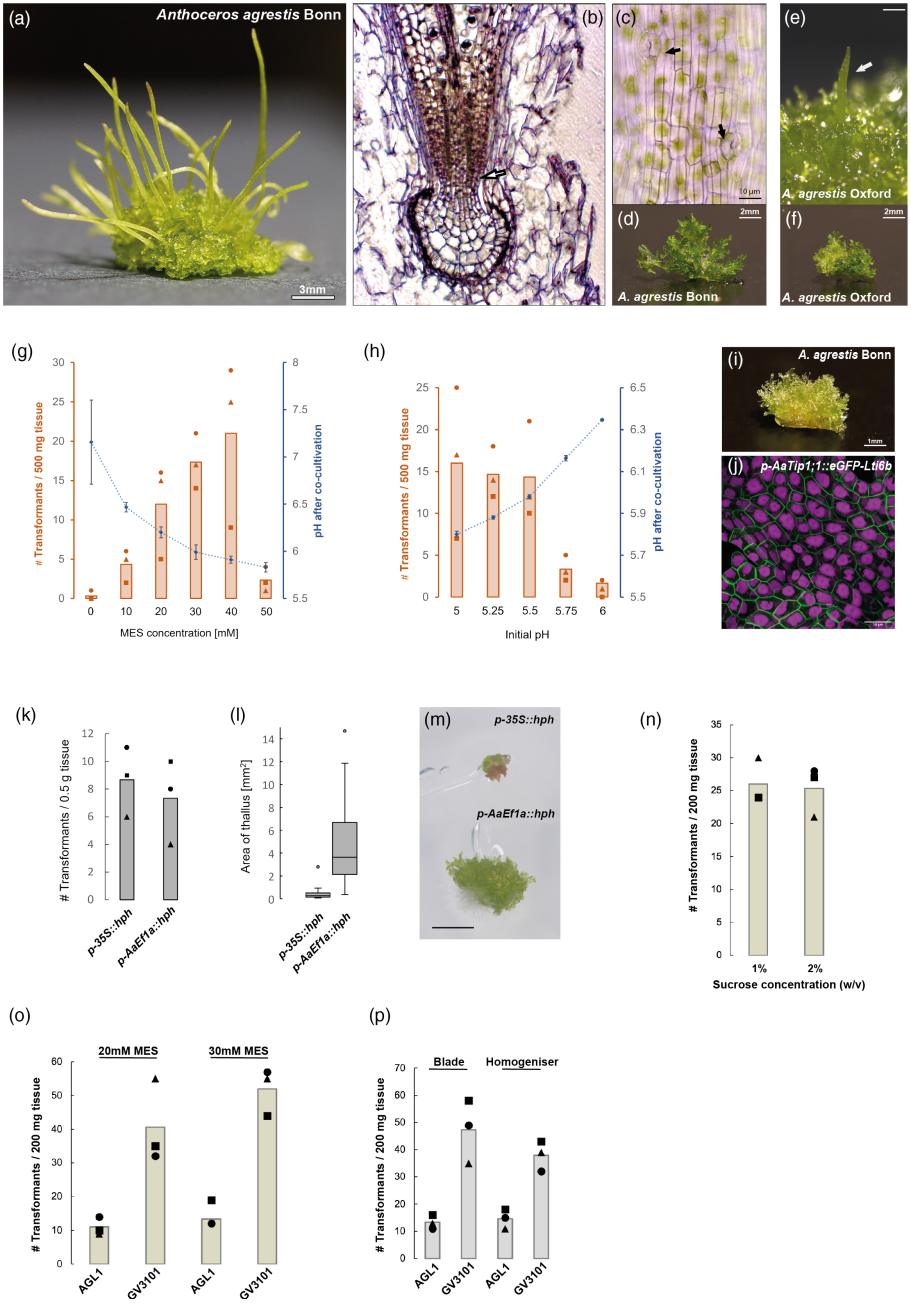
Characteristics of the *Anthoceros agrestis* Bonn strain and results of the optimization experiments to improve transformation efficiency. (a) *A. agrestis* Bonn plant bearing sporophytes. Scale bar: 3 mm. (b) Longitudinal section of the foot surrounded by the involucre showing the basal meristem (arrow) and differentiating sporogenous tissue. (c) Light microscopy image of the sporophyte epidermis showing stomata (see arrows). Scale bar: 10 μm. (d) Image of *A. agrestis* Bonn thallus fragment (before razor blade or ultra‐turrax aided fragmentation) used as starting material for the co‐cultivation with *Agrobacterium*. Scale bar: 2 mm. (e) *A. agrestis* Oxford plant bearing a young sporophyte. Scale bar: 3 mm. (f) Image of *A. agrestis* Oxford thallus fragment grown under the same conditions as the Bonn thallus in (d) (used as starting material for the co‐cultivation with *Agrobacterium*). Scale bar: 2 mm. (g, h) Comparison of the number of transformants (per 500 mg starting tissue) and final pH of the co‐cultivation media (after 3 days co‐cultivation) for (g) different MES concentrations in the co‐cultivation media (initial pH set to 5.5), and (h) different initial pH values of the co‐cultivation media with 40 mm MES. Graphs show values of triplicate experiments (symbols) and their average (bars). Error bars on pH values depicting SDs. (i) Transgenic *A. agrestis* Bonn transformed with the *p‐AaEf1a*::*hph* – *p‐AaTip1*;*1*::*eGFP‐Lti6b* construct. Scale bar: 1 mm. (j) Confocal microscopy image of *A. agrestis* Bonn gametophyte transformed with the *p‐AaEf1a*::*hph* – *p‐AaTip1*;*1*::*eGFP‐Lti6b* construct. Scale bar: 50 μm. Construct maps at Table S1. (k–m) Effect of the promoter (*AaEf1a* versus the CaMV 35S) driving the hygromycin selection cassette. (k) Histogram showing numbers of recovered independent transformant lines expressing GFP. (l) Boxplot showing distribution of thallus size between the two constructs (box depicts quartile and median), with whiskers showing maximum and minimum values inside 1.5× IQR, outliers outside that range depicted as dots. (m) Top: transgenic *A. agrestis* Bonn transformed with the *p‐35S*::*hph* – *p‐35S_s*::*eGFP‐Lti6b* construct. Bottom: transgenic *A. agrestis* Bonn transformed with the *p‐AaEf1a*::*hph* – *p‐AaTip1*;*1*::*eGFP‐Lti6b* construct. Scale bar: 1 mm. (n–p) Comparison of the number of transformants (per 200 mg starting tissue) (after 3 days co‐cultivation) for (n) different sucrose concentrations in the transformation buffer (initial pH set to 5.5, 20 mm MES), (o) different *Agrobacterium* strains used for the transformation (tested with *A. agrestis* Bonn), and (p) different fragmentation methods (tested with *A. agrestis* Bonn).

While the published transformation method was applied successfully to recover stable transformants for the Oxford isolate of *A. agrestis*, it resulted only in a very low number of stable transformants for the Bonn isolate. The Bonn isolate (Figure 1a–d) is of particular interest for embryo and sporophyte development studies, as, in contrast to the Oxford isolate (Figure 1e,f), sporophytes can be induced en masse under axenic conditions (Figure S1).

Our goal was to optimize the previously published transformation protocol for the *A. agrestis* Bonn strain and potentially apply it to other hornwort species. To do so, we examined a range of approaches and parameters that were most likely to influence the infection efficiency of *Agrobacterium* and the recovery of stable transformants: (i) the effect of the pH of the co‐cultivation media; (ii) the effect of the promoter driving the selection marker; (iii) the sucrose concentration of the co‐cultivation solution; and (iv) the choice of the *Agrobacterium* strain used. We successfully developed a modification of the Frangedakis, Waller, et al. (2021) protocol, that allowed us to obtain up to 55 stable, independent, transformant lines from 200 mg of tissue for the *A. agrestis* Bonn isolate and 103 stable transformant lines from 200 mg of tissue for the Oxford isolate.

We then tested whether the optimized transformation method is applicable to three other hornwort species: *Anthoceros punctatus*, *Leiosporoceros dussii*, and *Phaeoceros carolinianus. A. punctatus* has long been used as the study system for hornwort associations with cyanobacteria (*Nostoc punctiforme*) (Chatterjee et al., 2022). *L. dussii* is the sister taxon to all hornworts (Duff et al., 2007). *L. dussii* chloroplasts lack a pyrenoid and one of its key morphological innovations include a unique symbiotic arrangement of endophytic cyanobacteria (Villarreal et al., 2018; Villarreal & Renzaglia, 2006). *P. carolinianus* represents a commonly found species that can be grown relatively easily under laboratory conditions.

Finally, to facilitate future studies of hornworts at a cellular level, we developed a set of Loop cloning system/OpenPlant kit (Sauret‐Güeto et al., 2020) compatible tags for localization at the mitochondria, Golgi, peroxisomes, actin cytoskeleton, chloroplasts, and the endoplasmic reticulum (ER), and tested the utility of an additional fluorescent protein and chlorsulfuron as new selection marker for transformation.

In summary, we provide a streamlined transformation protocol, which can be used for a series of hornwort species and can potentially be applied to further species of hornworts.

## RESULTS

### Effect of 4‐morpholineethanesulfonic acid concentration on transformation efficiency

It has been reported that a stable pH of 5.5 during co‐cultivation leads to significantly increased *Agrobacterium* infection of *Arabidopsis thaliana* cells, likely due to inhibited calcium‐mediated defence signalling (Wang et al., 2018). As this innate immune response is possibly conserved among land plants, the pH during co‐cultivation may be a crucial factor for increased *Agrobacterium* infection rates of hornwort cells. While the published *A. agrestis* transformation method is employing phosphate‐buffered KNOP medium with pH 5.8 for co‐cultivation, monitoring pH during co‐cultivation of *A. agrestis* with *Agrobacterium*, revealed a pH value increase from an initial value of 5.8 to about 7–8, likely due to *Agrobacterium* growth. A test trial with the *p‐AaEf1a*::*hph* – *p‐AaTip1*;*1*::*eGFP‐Lti6b* construct (*AaEf1a*: *A. agrestis Elongation Factor 1a*, *hph*: hygromycin B resistance *phosphotransferase*, *AaTip1*;*1*: *A. agrestis Gamma Tonoplast Intrinsic Protein 1*;*1*, full list of all construct maps used in this study in Table S1), the initial pH set to 5.5 and the co‐cultivation medium supplemented with 10 mm 4‐morpholineethanesulfonic acid (MES) for improved buffering, increased the number of recovered stable transformants for *A. agrestis* Bonn. In this pre‐trial we also replaced the use of a homogenizer to fragment the thallus tissue (Figure S2, Video S1) before the co‐cultivation with a razor blade, to eliminate the need for special equipment.

To further test the influence of the pH range and stability during co‐cultivation on transformation efficiency, we added MES in a range of concentrations, 0–50 mm, to the co‐cultivation medium and evaluated the effect of different pH values on transformation efficiency. Increasing the concentration of MES in the co‐cultivation medium led to a slower rise of pH during co‐cultivation and greatly increased the number of transformants (Figure 1g). However, MES at concentrations higher than 40 mm had an apparent toxic effect on *A. agrestis* gametophyte tissue (Figure S3), as indicated by chloroplasts turning brown or grey, plant fragments immediately after co‐cultivation looked generally less healthy compared with plants co‐cultivated using <40 mm MES concentrations. When applying an MES concentration of 50 mm, most plant fragments appeared to be dead within 4–5 days after co‐cultivation, leading to a decrease in the total number of recovered transformants. The optimal MES concentration was therefore determined to be 40 mm MES.

### Effect of initial pH of co‐cultivation media on transformation efficiency

We also tested the effect of the initial pH of the co‐cultivation medium on transformation efficiency. Comparison of the different initial pH from 5 to 5.5 showed no significant difference in transformation efficiency, but the number of transformants decreased when the starting pH was set to ≥5.75 (Figure 1h–j). In conclusion, the highest number of stable transformants could be recovered with an initial pH between 5 and 5.5 and 40 mm MES for improved buffering. It should be noted that this is only valid for the specific tissue culture conditions used in our trials, and using thallus fragments that have been propagated for up to 6 weeks after subculturing (Figure S3). Non‐empirical observations suggest that thallus tissue from older cultures (2–3 months after subculturing) show a higher sensitivity to MES, with 20 mm MES frequently killing many cells. In this case, higher numbers of recovered stable transformants were usually obtained with only 10 mm MES.

### Effect of promoter on selection and recovery of transformant lines

The promoter used to drive expression of the selection gene hygromycin B resistance‐encoding *hph* may influence the number of recovered stable transformants. The cauliflower‐mosaic virus (CaMV) 35S promoter previously used for this purpose shows stronger expression in differentiated cells of the thallus tissue and weaker expression in new grown parts of the thalli (Frangedakis, Waller, et al., 2021). In contrast, the endogenous *AaEf1a* promoter is more strongly expressed in new grown tissue of the Oxford isolate (Frangedakis, Waller, et al., 2021), which we hypothesize to be more susceptible to *Agrobacterium* infection. Interestingly, the *AaEf1a* promoter shows high activity in protoplasts of *A. agrestis* Bonn, while CaMV 35S activity could not be detected (Neubauer et al., 2022). Thus, we reasoned that driving expression of the *hph* under the *AaEf1a* promoter might improve transformation efficiency.

To test the effect on transformation efficiency when driving the expression of the hygromycin resistance (*hph*) gene with either the *AaEf1a* or the CaMV 35S promoter, we transformed equal amounts of *A. agrestis* Bonn tissue with two different vectors. One contained a *p‐35S*::*hph* transcription unit and the other a *p‐AaEf1a*::*hph* transcription unit. Both vectors further contained a *p‐AaTip1*;*1*::*eGFP‐Lti6b* transcription unit to allow the use of fluorescence as an additional marker for successful transformation. We then compared the number of isolated enhanced green fluorescent protein (eGFP) expressing transformants and their growth after a total of 7 weeks on selective media. For both constructs, the number of primary transformants (thalli) expressing eGFP in at least some cells of the thallus was comparable (Figure 1k). However, the majority of plants carrying the *hph* gene driven by the CaMV 35S promoter showed retarded growth compared with plants carrying the *hph* gene driven by the *AaEf1a* promoter. In particular, after 7 weeks of growth, the surface area of thalli of transformants carrying the *p‐AaEf1a*::*hph* transcription unit, was on average 10 times that of plants carrying the *p‐35S*::*hph* transcription unit (Figure 1l,m) (based on three independent experimental replicates). Therefore, while the number of initially isolated transformant lines is not affected by the choice of the promoter driving *hph*, it does have a large effect on the recovery and growth rate of transformed thalli. Different ‘enhanced’ versions of the CaMV 35S promoter might be more effective in driving selection markers in the Bonn strain.

### Effect of sucrose concentration of the co‐cultivation medium on transformation efficiency

We compared the effect on transformation efficiency of two different concentrations of sucrose, 1% (w/v) and 2% (w/v). We reasoned that lowering the concentration of sucrose in the co‐cultivation medium might reduce the chances of *Agrobacterium* overgrowth during co‐cultivation that can potentially affect the pH of the medium and consequently transformation efficiency. We infected equal amounts of *A. agrestis* Bonn tissue using co‐cultivation media with 20 mm MES, the *AGL1 Agrobacterium* harbouring the *p‐AaEf1a*::*hph* – *p‐35S_s*::*eGFP‐Liti6b* construct and either 1% (w/v) or 2% (w/v) sucrose concentration. Our results suggest that the transformation efficiency is comparable (Figure 1n).

### Effect of *Agrobacterium* strain used

Previously, only a very small number of transformants were obtained when the *GV3101 Agrobacterium* strain was used. We tested again the *GV3101 Agrobacterium* strain for its ability to infect and transform *A. agrestis* thallus compared with the *AGL1* strain. We infected equal amounts of *A. agrestis* Bonn and Oxford tissue using co‐cultivation media with 20 mm MES with *GV3101* or *AGL1 Agrobacterium* harbouring the *p‐AaEf1a*::*hph* – *p‐AaEf1a*::*eGFP‐Liti6b* construct. We repeated the same experiment using co‐cultivation media with 20 and 30 mm MES concentrations. Our results revealed that when the *GV3101* strain was used the number of transformants was greater (Figure 1o). This contradicts our previous findings (Frangedakis, Waller, et al., 2021), which could be explained by differences in the quality of *GV3101 Agrobacterium* batches, and/or the difference in pH stability during co‐cultivation.

### Comparison of homogenized versus blade fragmented tissue

To test the effect of the tissue fragmentation method on transformation efficiency we infected equal amounts of *A. agrestis* Bonn tissue fragmented using a razor blade or a homogenizer and using co‐cultivation media with 30 mm MES and the *AGL1* or *GV3101 Agrobacterium* harbouring the *p‐AaEf1a*::*hph* – *p‐AaEf1a*::*eGFP‐Liti6b* construct. Our results show that using a razor blade instead of a homogenizer does not significantly affect overall transformation efficiency. This indicates that fragmentation can be carried out without the need of specialized equipment (Figure 1p).

### Comparison of transformation efficiency between the two *Anthoceros agrestis* isolates and *Anthoceros punctatus*

**Figure 2:**
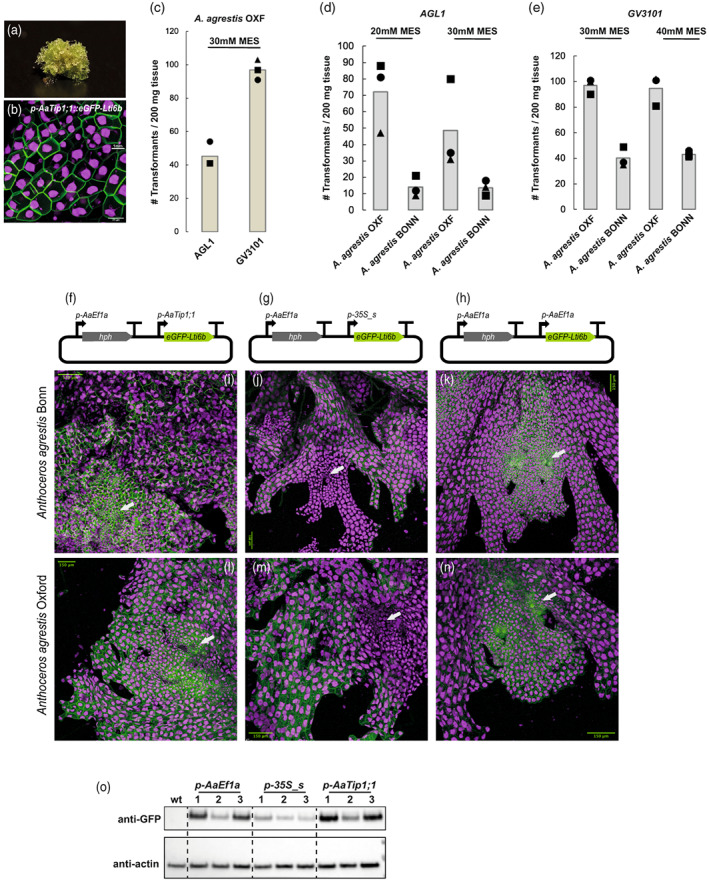
Results of transformation efficiency optimization experiments for the *Anthoceros agrestis* isolates, schematic representation of transformation constructs, and confocal microscopy images of transgenic *A. agrestis* isolates. (a) Transgenic *A. agrestis* Oxford gametophyte transformed with the *p‐AaEf1a*::*hph* – *p‐AaTip1*;*1*::*eGFP‐Lti6b* construct. Scale bar: 1 mm. (b) Confocal microscopy image of *A. agrestis* Oxford gametophyte transformed with the *p‐AaEf1a*::*hph* – *p‐AaTip1*;*1*::*eGFP‐Lti6b* construct. Scale bar: 25 μm. (c) Comparison of the number of transformants (per 200 mg starting tissue) (after 3 days co‐cultivation) for *A. agrestis* Oxford isolate using two different *Agrobacterium* strains (*AGL1* and *GV3101*). (d) Comparison of number of transformants (per 200 mg starting tissue) (after 3 days co‐cultivation) for the two *A. agrestis* isolates under two different MES concentrations in the transformation buffer (initial pH set to 5.5) using the *AGL1 Agrobacterium* strain. (e) Comparison of number of transformants (per 200 mg starting tissue) (after 3 days co‐cultivation) for the two *A. agrestis* isolates under two different MES concentrations in the transformation buffer (initial pH set to 5.5 using the *GV3101 Agrobacterium* strain). (f–h) Schematic representation of constructs for the expression of two transcription units (TU): one TU for the expression of the *hygromycin B phosphotransferase* (*hph*) gene under the control of the *AaEf1a* promoter, and one TU for the expression of (f) *p‐AaEf1a*::*hph* – *p‐AaTip1*;*1*::*eGFP‐Lti6b*. (g) *p‐AaEf1a*::*hph* – *p‐35S_s*::*eGFP‐Lti6b*. (h) *p‐AaEf1a*::*hph* – *p‐AaEf1a*::*eGFP‐Lti6b*. (i–n) Confocal images of transformed lines: (i) *A. agrestis* Bonn transformed with the *p‐AaEf1a*::*hph* – *p‐AaTip1*;*1*::*eGFP‐Lti6b* construct. (j) *A. agrestis* Bonn transformed with the *p‐AaEf1a*::*hph* – *p‐35S_s*::*eGFP‐Lti6b* construct. (k) *A. agrestis* Bonn transformed with the *p‐AaEf1a*::*hph* – *p‐AaEf1a*::*eGFP‐Lti6b* construct. (l) *A. agrestis* Oxford transformed with the *p‐AaEf1a*::*hph* – *p‐AaTip1*;*1*::*eGFP‐Lti6bP* construct. (m) *A. agrestis* Oxford transformed with the *p‐AaEf1a*::*hph – p‐35S_s*::*eGFP‐Lti6b* construct. (n) *A. agrestis* Oxford transformed with the *p‐AaEf1a*::*hph – p‐AaEf1a*::*eGFP‐Lti6b* construct. Apical cells of the thallus margin and the surrounding tissue are marked with white arrows. Scale bars: 150 μm. All construct maps can be found in Table S1. (o) Western blot analysis of eGFP accumulation in transgenic lines. Total cellular proteins were separated by polyacrylamide gel electrophoresis, blotted, and the probed with anti‐GFP and anti‐actin antibodies. Numbering above the blot images corresponds to the identifiers of independent transformed lines.

We then confirmed that the protocol can successfully be used to recover stable transformants for the *A. agrestis* Oxford isolate (Figure 2a,b).

To estimate the transformation efficiency of the protocol for the two *A. agrestis* isolates (Bonn and Oxford), we performed transformation trials using 200 mg of tissue per trial, co‐cultivation media with 20, 30, or 40 mm MES, *AGL1* or *GV3101 Agrobacterium* harbouring the *p‐AaEf1a*::*hph* – *p‐AaEf1a*::*eGFP‐Lti6b* construct. The number of successful transformants (plant thalli) per experiment is summarized in Figure 2c,e. We could recover up to three times more transformants for the Oxford isolate compared with the Bonn isolate.

### Characterization of the activity of the *AaTip1* ;*1* promoter

Previously, a 1368 bp putative promoter fragment of the *A. thaliana Tip1*;*1* gene homologue in *A. agrestis* was selected as a candidate for a constitutive hornwort promoter (Frangedakis, Waller, et al., 2021). Here we further characterize the *AaTip1*;*1* promoter and compared it with the two other constitutive, *AaEf1a* and the CaMV 35S, promoters used for *A. agrestis*.

Expression of eGFP driven by the *AaTip1*;*1* promoter is equally strong across the thallus (Fig 2f–n). As reported earlier (Frangedakis, Waller, et al., 2021), expression of eGFP driven by the CaMV 35S promoter seems to be weaker in meristematic areas of the thallus (Figure 2f–n), unlike expression of eGFP driven by the *AaEf1a* promoter, which is stronger in meristematic areas of the thallus. It must be noted that the eGFP expression in the stable transformants can be categorized into four groups: expression throughout the thallus, expression in the rhizoids, expression in patches, or no expression (Figure S4). Frequency of each of those four events is summarized in Table 1.

**Table 1:** eGFP expression patterns in the stable transformants

Construct	Species/strain	Number of transformants	Expression in rhizoids	Patchy expression	No fluorescence
*p‐AaEf1a*::*hph* – *p‐AaEf1a*::*eGFP‐Lti6b*	Oxford	211	22	13	10
*p‐AaEf1a*::*hph* – *p‐AaEf1a*::*eGFP‐Lti6b*	Bonn	78	17	8	7
*p‐AaEf1a*::*hph* – *p‐AaEf1a*::*eGFP‐Lti6b*	Bonn GV3101	122	22	15	11
*p‐AaEf1a*::*hph* – *p‐AaEf1a*::*eGFP‐Lti6b*	*A. punctatus*	28	5	4	1
*p‐AaEf1a*::*hph* – *p‐35S_s*::*eGFP‐Lti6b*	Bonn	8	1	‐	3
*p‐AaEf1a*::*hph* – *p‐35S_s*::*eGFP‐Lti6b*	Oxford	146	21	10	7
*p‐AaEf1a*::*hph* – *p‐AaTip1*;*1*::*eGFP‐Lti6b*	Bonn	34	10	10	5
*p‐AaEf1a*::*hph* – *p‐AaTip1*;*1*::*eGFP‐Lti6b*	Oxford	16	1	4	1
*p‐AaEf1a*::*hph* – *p‐AaTip1*;*1*::*eGFP‐Lti6b*	*A. punctatus*	7	–	–	2

We also compared the amount of eGFP protein accumulated in plants expressing eGFP under the *AaTip1*;*1*, the CaMV 35S or the *AaEf1a* promoter. Western blot analysis showed that the *AaTip1*;*1* and the *AaEf1a* promoters lead to similar abundances of fluorescent proteins at the whole thallus level. By contrast, the CaMV 35S promoter was weaker (Figure 2o).

**Figure 3:**
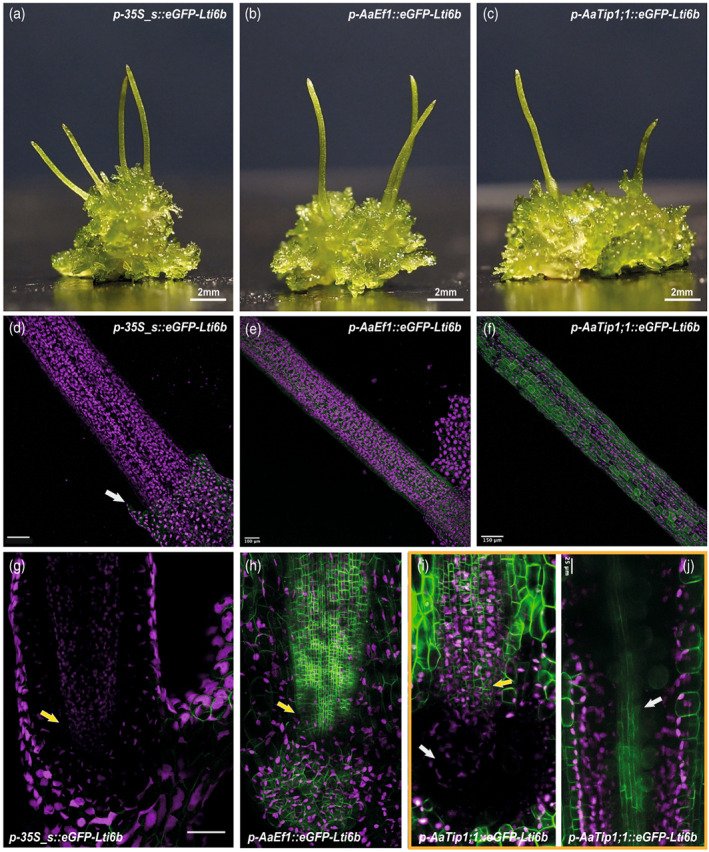
Promoter activity in the *Anthoceros agrestis* Bonn sporophyte. (a) Transgenic *A. agrestis* Bonn with sporophytes transformed with the *p‐AaEf1a*::*hph* – *p‐35S_s*::*eGFP‐Lti6b* construct. (b) Transgenic *A. agrestis* Bonn with sporophytes transformed with the *p‐AaEf1a*::*hph* – *p‐AaEf1a*::*eGFP‐Lti6b* construct. (c) Transgenic *A. agrestis* Bonn with sporophytes transformed with the *p‐AaEf1a*::*hph* – *p‐AaTip1*;*1*::*eGFP‐Lti6b* construct. (d–f) Confocal microscopy images (whole‐mount) of *A. agrestis* Bonn sporophytes transformed with (d) the *p‐AaEf1a*::*hph* – *p‐35S_s*::*eGFP‐Lti6b* construct. Scale bar: 150 μm. (e) *p‐AaEf1a*::*hph* – *p‐AaEf1a*::*eGFP‐Lti6b* construct. Scale bar: 100 μm (f) *p‐AaEf1a*::*hph* – *p‐AaTip1*;*1*::*eGFP‐Lti6b* construct. Scale bar: 150 μm. (g) Confocal microscopy images of the sporophyte base of *A. agrestis* Bonn transformed with the *p‐AaEf1a*::*hph* – *p‐35S_s*::*eGFP‐Lti6b* construct, basal meristem indicated with white arrow. Scale bar: 100 μm. (h) Confocal microscopy images of the sporophyte base of *A. agrestis* Bonn transformed with the *p‐AaEf1a*::*hph* – *p‐AaEf1a*::*eGFP‐Lti6b*, basal meristem indicated with white arrow. Scale bar: 100 μm. (i,j) Multiphoton microscopy images (whole mount) of the sporophyte base of *A. agrestis* Bonn transformed with the *p‐AaEf1a*::*hph* – *p‐AaTip1*;*1*::*eGFP‐Lti6b* construct. (i) no eGFP signal is visible in the foot (white arrow), but in overlying cell rows of the basal meristem (yellow arrow). (j) eGFP signal is strongest in the epidermal cells and the columella (white arrow). Scale bar: 25 μm. All construct maps can be found in Table S1.

We also tested the expression of the three constitutive promoters at the sporophyte stage of *A. agrestis* Bonn. When eGFP was driven by the CaMV 35S promoter no eGFP fluorescence could be observed in the sporophyte (Figure 3a,d,g). In contrast, when eGFP expression was driven by the *AaEf1a* or the *AaTip1*;*1* promoter, eGFP fluorescence was detectable in most sporophyte tissues (Figure 3b,c,e,f,h,i,j).

### The protocol can be used to transform more hornwort species

**Figure 4:**
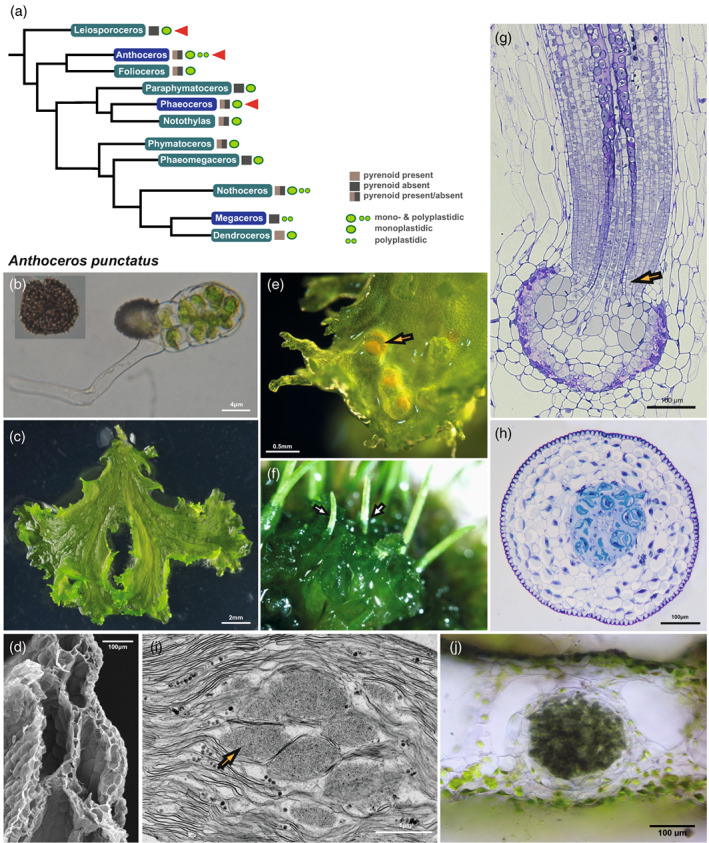
Hornwort phylogeny and the morphological features of *Anthoceros punctatus*. (a) Hornwort phylogeny (Villarreal & Renner, 2012). Genera whose species could be successfully transformed using our improved protocol are marked with red arrowheads. (b–j) Morphology of *A. punctatus*. (b) Germinating spore that develops into a thallus. Scale bar: 4 μm. (c) The thallus is irregularly shaped, lacks specialized internal differentiation and is composed of mucilage chambers and parenchyma cells. Scale bar: 2 mm. (d) Scanning electron microscopy of thallus. Scale bar: 100 μm. The male (antheridia are shown with a yellow arrow) (e) and female (archegonia) reproductive organs are embedded in the thallus. Scale bar: 0.5 mm. (f) Thallus with sporophytes (see white arrow). (g) Longitudinal section of the foot (see yellow arrow) surrounded by the involucre showing the basal meristem and differentiating sporogenous tissue. Scale bar: 100 μm. (h) Transverse section of the sporophyte showing its morphology. From the centre to outside: columella, spores, pseudoelaters, assimilative tissue, epidermis, and stomata with substomatal cavities. Scale bar: 100 μm. (i) Transmission electron microscopy of the chloroplast (yellow arrow: pyrenoid). Scale bar: 1 μm. (j) Hand section of *A. punctatus* thallus showing ellipsoidal cavity colonized by cyanobacteria. Scale bar: 100 μm.

Hornworts comprise 11 genera (Villarreal & Renner, 2012). which include: *Leiosporoceros*, *Anthoceros*, *Folioceros*, *Paraphymatoceros*, *Phaeoceros*, *Notothylas*, *Phymatoceros*, *Phaeomegaceros*, *Nothoceros*, *Megaceros*, and *Dendroceros*. (Figure 4a). We tested whether the optimized protocol for the *A. agresis* isolates can successfully be used to obtain transgenic *A. punctatus* (the model for cyanobacteria symbiosis studies), *L. dussii* (sister to all other hornworts), and *P. carolinianus* plants.

*A. punctatus*, as *A. agrestis*, can routinely be propagated vegetatively in lab conditions, with monthly subculturing. Its genome is sequenced and has a size of approximately 132 Mbp. However, sexual reproduction under lab conditions has been challenging. Spores are punctuated (Figure 4b). The *A. punctatus* thallus is irregularly shaped (Figure 4c), lacks specialized internal differentiation and is composed of mucilage chambers and parenchyma cells (Figure 4d). The male (Figure 4e) and female reproductive organs are embedded in the thallus. Sporophytes grow on the gametophyte (Figure 4f) and contain columella, spores, pseudoelaters, assimilative tissue, epidermis, and stomata (Figure 4g,h). *A. punctatus* chloroplasts have pyrenoids (Figure 4i) and its thallus has ellipsoidal cavities colonized by cyanobacteria (Figure 4j).

**Figure 5:**
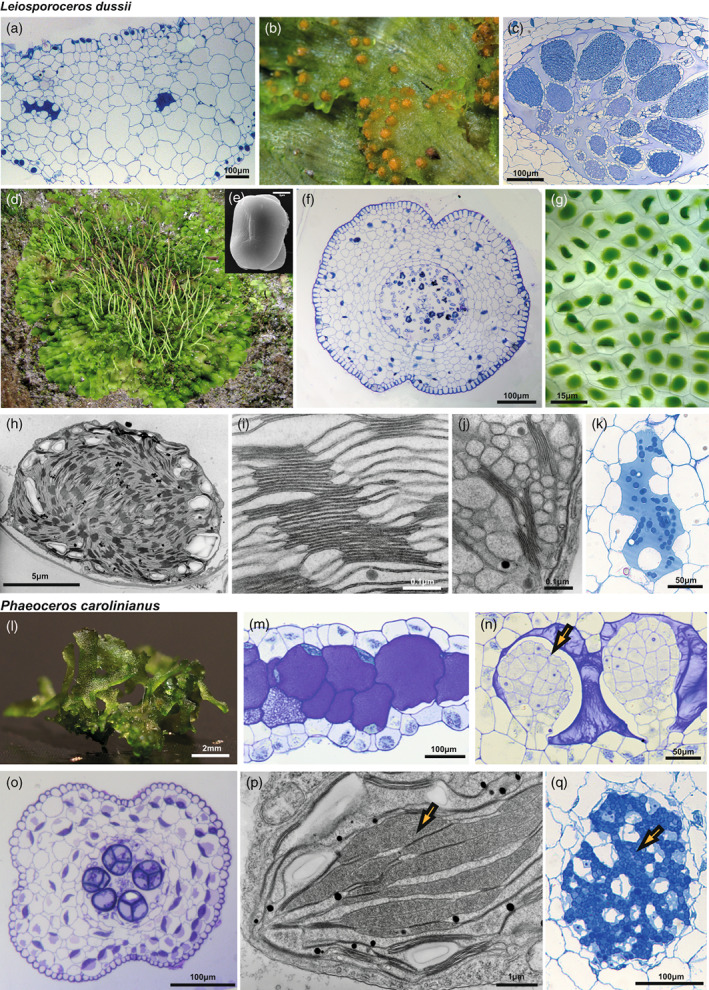
Morphology of *Leiosporoceros dussii* and *Phaeoceros carolinianus*. (a–k) Morphology of *L. dussii*. (a) *L. dussii* thallus is irregularly shaped, lacks specialized internal differentiation and is composed of mucilage chambers and parenchyma cells. Scale bar: 100 μm. (b) *L. dussii* plant with antheridia. (c) Light microscopy section showing antheridia embedded in thallus. Scale bar: 100 μm. (d) *L. dussii* with sporophytes. (e) Scanning electron microscopy of spore. Unlike other hornworts it is monolete and smooth. Scale bar: 5 μm. (f) Transverse section of the sporophyte showing its morphology. From the centre to outside: columella, spores, pseudoelaters, assimilative tissue, epidermis, and stomata with substomatal cavities. Scale bar: 100 μm. (g) Light microscopy image of *L. dussii* gametophyte showing a single chloroplast per cell. Scale bar: 15 μm. (h–j) Transmission electron microscopy of chloroplast. Scale bars: (h) 5 μm, (i) 0.1 μm, and (j) 0.1 μm. (k) Light microscopy section of *Nostoc* colony showing algal cells. Scale bar: 50 μm. (l–q) Morphology of *P. carolinianus*. (l) *P. carolinianus* gametophyte grown under laboratory conditions. Scale bar: 2 mm. (m) Thallus with mucilage cells. Scale bar: 100 μm. (n) Light microscopy section showing antheridia (yellow arrow) embedded in thallus. Scale bar: 50 μm. (o) Transverse section of the sporophyte showing its morphology. From the centre to outside: columella, spores, pseudoelaters, assimilative tissue, epidermis, and stomata with substomatal cavities. Scale bar: 100 μm. (p) Transmission electron microscopy of chloroplast (yellow arrow pointing to the pyrenoid). Scale bar: 1 μm. (q) Light microscopy section of *Nostoc* colony showing algal cells. Scale bar: 100 μm (yellow arrow pointing to the cyanobacteria).

*L. dussii* typically has a solid thallus with schizogenous cavities (Figure 5a). Antheridia are numerous (Figure 5b,c) and sporophytes (Figure 5d) develop on the gametophyte. Unlike other hornworts, spores are monolete not trilete and smooth (Figure 5e). The sporophyte (Figure 5f) is consisting of the columella, the sporogenous tissue, the assimilative layer, epidermis, and stomata. *L. dussii* has a single chloroplast per cell that lacks a pyrenoid (Figure 5g,h). Chloroplasts have numerous channel thylakoids and extensive grana stacks (Figure 5i,j). The thallus of *L. dussii* is colonized with *Nostoc* cyanobacteria (Figure 5k), which are longitudinally oriented strands in mucilage‐filled schizogenous canals.

The *P. carolinianus* thallus is irregularly shaped (Figure 5l) and lacks specialized internal differentiation (Figure 5m). *P. carolinianus* is monoicous and produces both antheridia (Figure 5n) and archegonia on the dorsal side of the thallus. The sporophyte (Figure 5o) consists of the columella, sporogenous tissue, assimilative layer, epidermis, and stomata. *P. carolinianus* has a single chloroplast per cell with pyrenoids (Figure 5p) and its thallus is colonized by cyanobacteria (Figure 5q).

**Figure 6:**
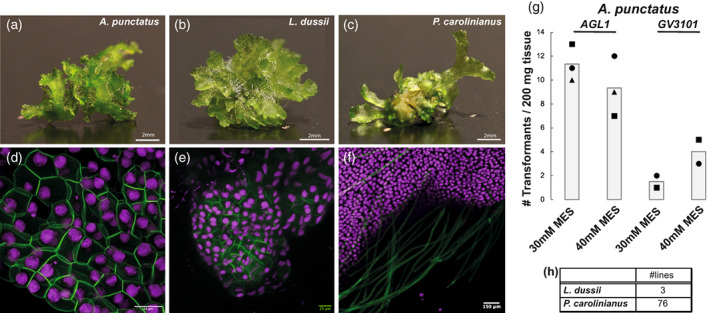
Confocal microscopy images of transgenic *Anthoceros punctatus*, *Leiosporoceros dussii*, and *Phaeoceros carolinianus*. (a) *A. punctatus*, (b) *L. dussii*, and (c) *P. carolinianus* thallus fragments used as starting material for co‐cultivation with *Agrobacterium*. Scale bars: 2 mm. Confocal microscopy images. (d) *A. punctatus*. Scale bar: 50 μm. (e) *L. dussii* and (f) *P. carolinianus* gametophytes transformed with the (d, f) *p‐AaEf1a*::*hph* – *p‐AaTip1*;*1*::*eGFP‐Lti6b* and (e) *p‐AaEf1a*::*hph* – *p‐AaEf1a*::*eGFP‐Lti6b* construct. Scale bar: 150 μm. (g) Comparison of number of transformants (per 200 mg starting tissue) (after 3 days co‐cultivation) for *A. punctatus* under two different MES concentrations in the transformation buffer (initial pH set to 5.5) using the *AGL1* or *GV3101 Agrobacterium* strain. (h) Total number of stable lines obtained for *L. dussii* and *P. carolinianus*.

We performed preliminary trials using as starting material 200 mg of tissue per trial, co‐cultivation media with 30 or 40 mm MES, *AGL1 Agrobacterium* harbouring the *p‐AaEf1a*::*hph* – *p‐AaEf1a*::*eGFP‐Lti6b* plasmid. We successfully recovered stable transformants for all three species (Figure 6a–f). We also performed trials for the three species using 200 mg of tissue per trial, co‐cultivation media with 40 mm MES and the *GV3101 Agrobacterium* harbouring the *p‐AaEf1a*::*hph* – *p‐35S_s*::*eGFP‐Lti6b* plasmid; however, we only recovered transformants for *A. punctatus*. The number of successful transformants (plant thalli) per experiment are summarized in Figure 6g,h. *A. punctatus* has the highest transformation efficiency and *L. dussii* has the lowest efficiency with only three stable lines recovered in total.

### Simplified new transformation method

**Figure 7:**
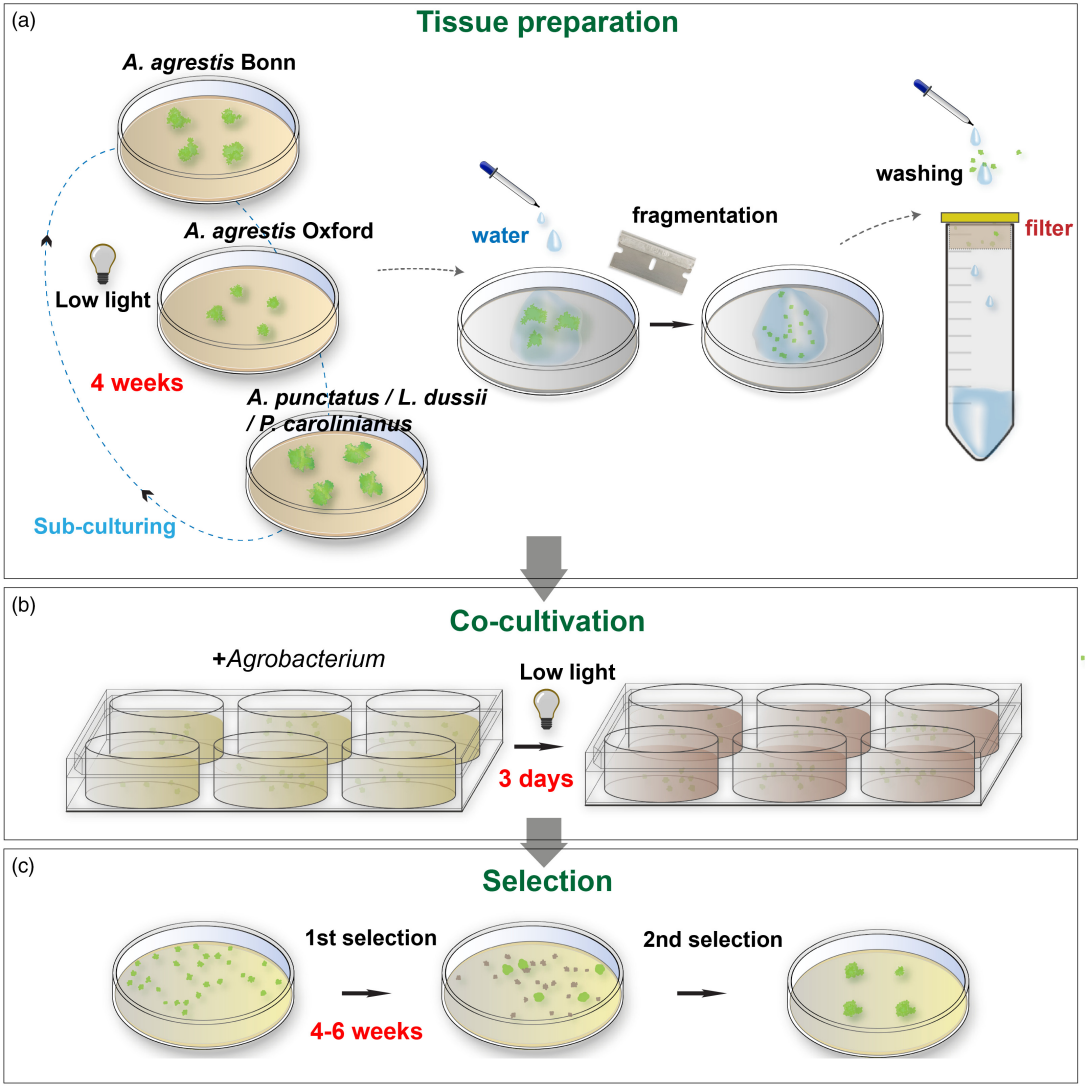
Schematic representation of the optimized transformation protocol. (a) Thallus tissue is routinely propagated on a monthly basis under low light. Four‐ to 6‐week‐old tissue is fragmented with the aid of a razor blade, transferred to a cell strainer, and washed thoroughly with sterile water. (b) The tissue is then co‐cultivated with *Agrobacterium* for 3 days (under low light) and (c) spread on antibiotic‐containing growth medium. After approximately 4–6 weeks, putative transformants are visible. A final round of selection is used to eliminate false‐positive transformants.

In summary the steps of the new optimized protocols are as follows (Figure 7, detailed description is provided in Methods and Figures S5–S8): fragmented regenerating thallus tissue (grown for at least 4 weeks) was co‐cultivated with *Agrobacterium* in liquid KNOP supplemented with 1% (w/v) sucrose medium. Liquid media were supplemented with 40 mm MES and 3,5‐dimethoxy‐4‐hydroxyacetophenone (acetosyringone) at a final concentration of 100 μm. The pH was adjusted to 5.5. Co‐cultivation duration was 3 days with shaking at 21°C on a shaker without any light supplementation (only ambient light from the room). After co‐cultivation the tissue was plated on solid KNOP plates supplemented with 100 μg ml^−1^ cefotaxime and 10 μg ml^−1^ hygromycin. A month later successful transformants based on rhizoid production were visible on the plate. After 4 weeks it is recommended to transfer the tissue on fresh selective media plates. As we have demonstrated previously, the transgene is stably integrated into the genome (Frangedakis, Waller, et al., 2021) and lines can be propagated for years without losing the transgene.

### New tools to label and target specific compartments of the hornwort cell

**Figure 8:**
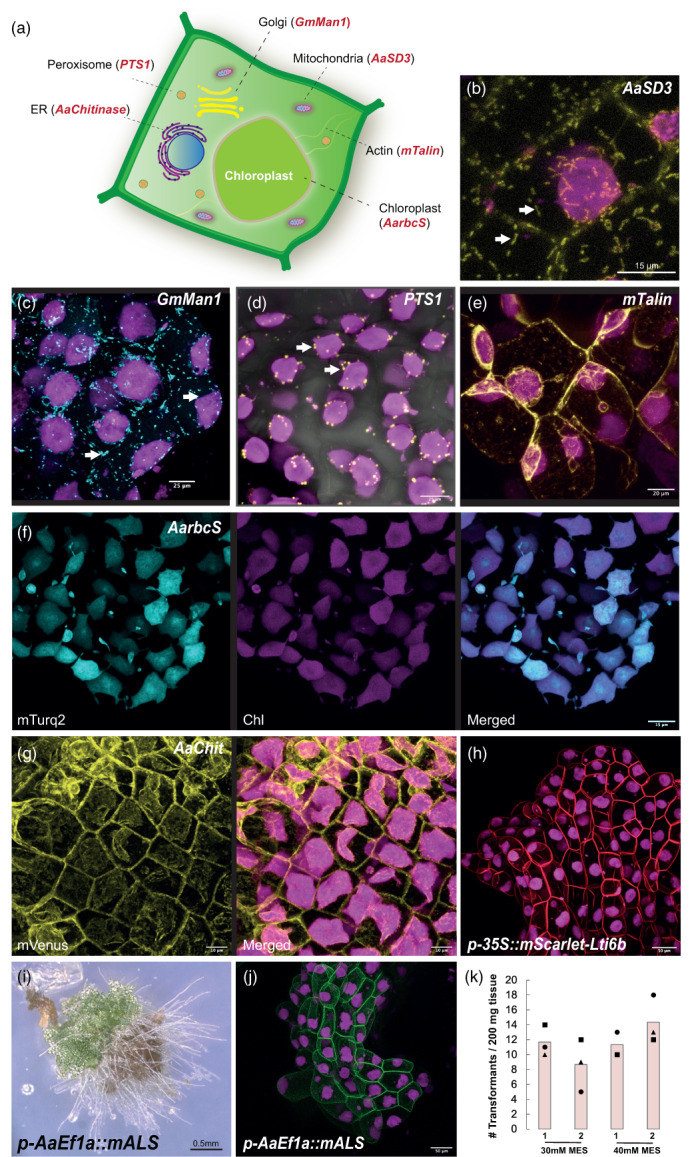
Targeting various subcellular components of the *Anthoceros agrestis* thallus with different localization tags. (a) Schematic representation of a hypothetical *A. agrestis* cell showing a summary of the new subcellular localization peptides developed in this study. (b) Confocal microscopy image of *A. agrestis* Oxford expressing the *p‐AaEf1a*::*hph* – *p‐AaEf1a*::*mVenus‐AaSD3* construct. Scale bar: 15 μm. (c) Confocal microscopy image of *A. agrestis* Oxford expressing the *p‐AaEf1a*::*hph* – *p‐AaEf1a*::*mTurquoise2‐GmMan1* construct. Scale bar: 25 μm. (d) Confocal microscopy image of *A. agrestis* Oxford expressing the *p‐35S*::*hph* – *p‐35Sx2*::*mVenus‐PTS1* construct. Scale bar: 25 μm. (e) Confocal microscopy image of *A. agrestis* Oxford expressing the *p‐35S*::*hph* – *p‐35S*::*mVenus‐mTalin* construct. Scale bar: 20 μm. (f) Confocal microscopy image of *A. agrestis* Oxford expressing the *p‐AaEf1a*::*hph* – *p‐AaEf1a*::*AarbcS‐mTurquoise* construct. Scale bar: 15 μm. (g) Confocal microscopy image of *A. agrestis* Oxford expressing the *p‐AaEf1a*::*hph* – *p‐AaEf1a*::*mVenus‐AaChit* construct. Scale bar: 10 μm. (h) Confocal microscopy image of *A. agrestis* Oxford expressing the *p‐AaEf1a*::*hph* – *p‐35S*::*mScarlet‐Lti6b* construct. Scale bar: 50 μm. (i) Light microscopy image of 6‐week‐old *A. agrestis* Bonn regenerating fragment transformed with the *p‐AaEf1a*::*mALS* – *p‐35S_s*::*eGFP‐Lti6b* construct. Scale bar: 0.50 m. (j) Confocal microscopy image of *A. agrestis* Bonn expressing the *p‐AaEf1a*::*mALS* – *p‐35S_s*::*eGFP‐Lti6b* construct. Scale bar: 50 μm. (k) Comparison of number of transformants (per 200 mg starting tissue) (after 3 days co‐cultivation) for *A. agrestis* Bonn under two different MES concentrations in the transformation buffer (initial pH set to 5.5) using the *AGL1* or *GV3101 Agrobacterium* strain harbouring the *p‐AaEf1a*::*mALS* – *p‐35S_s*::*eGFP‐Lti6b* construct.

Hornwort cell biology is poorly understood. While other bryophytes such as the moss *Physcomitrium patens* and the liverwort *M. polymorpha* are being used increasingly to study cellular processes, to what extent these findings can be extended to hornworts is unclear due to their independent evolutionary history spanning over 400 million years (Naramoto et al., 2022; Pfeifer et al., 2022). Consequently, developing tools for hornwort specific cell biology studies is necessary. Hornworts also have multiple unique cellular features absent in mosses, liverworts, or vascular plants awaiting to be investigated. For instance, monoplastidic cells, putative plastid stromules, and the presence/absence of a carbon concentrating structure in the chloroplast known as the pyrenoid. More generally, hornworts can be useful experimental systems to understand fundamental aspects of plant cell biology such as cell polarity, plasmodesma‐related processes, and cell division. This is thanks to the simple morphology and the relatively flat shape of hornwort thallus that makes imaging at the cellular level easier compared with other systems. Thus, to develop tools facilitating the study of hornworts at a cellular level, we tested a series of potential signal peptides for subcellular localization at the mitochondria, Golgi, peroxisome, actin cytoskeleton, chloroplast, and the ER (Figure 8a; Figure S9). We further tested the applicability in hornworts, of new fluorescent proteins, a different reporter, and a new selection marker.

Mitochondria in the liverwort, *M. polymorpha*, have been visualized using a targeting sequence derived from the *A. thaliana Segregation Distortion 3* (*SD3*) gene, which encodes a protein with high similarity to the yeast translocase of the inner mitochondrial membrane 21 (TIM21) (Ogasawara et al., 2013). A mitochondrial targeting sequence from the *Saccharomyces cerevisiae COX4* (*ScCOX4*) gene (Hamasaki et al., 2012) can also be used successfully in *M. polymorpha* (Figure S10). However, mitochondrial localization was not achieved when using either the *A. thaliana SD3* or the *ScCOX4* targeting sequences in *A. agrestis* Oxford. We thus tested the N‐terminal sequence of a predicted *SD3 A. agrestis* gene (Sc2ySwM_117.1379.1) (Figure S9), for its ability to direct fluorescent localization in mitochondria. Using this sequence (*p‐AaEf1a*::*hph* – *AaEf1a*::*mVenus‐AaSD3* construct), we observed fluorescence in structures in the cytosol that resemble mitochondria (Figure 8b). To confirm that these structures are indeed mitochondria, we stained thallus fragments with MitoTracker. The signal from mVenus overlapped with that from MitoTracker (Figure S11a–c), demonstrating that the N‐terminal sequence of *AaSD3* is sufficient to visualize mitochondria. We note that the observed signal has low fluorescence intensity and the use of a confocal microscope at high magnification is necessary for observation.

In *M. polymorpha* the Golgi has been visualized using the transmembrane domain of the rat sialyltransferase (Figure S10) as a targeting sequence (Kanazawa et al., 2016). Another Golgi localization sequence, which can be used in *M. polymorpha,* is a targeting sequence derived from the soybean (*Glycine max*) *α‐1*,*2 mannosidase 1* (*GmMan1*) gene (Luo & Nakata, 2012) (Figure S9). We tested whether the sialyltransferase or the *GmMan1* peptides can be successfully used in *A. agrestis* Oxford for Golgi targeting. Only the *GmMan1* peptide (*p‐AaEf1a*::*hph* – *p‐35Sx2*::*mTurquoise2‐GmMan1* construct) led to fluorescence in structures in the cytosol that resemble Golgi (Figure 8c).

Previous studies in *M. polymorpha* have used the peroxisomal targeting signal 1 (PTS1) (Ser‐Lys‐Leu) as a peroxisome targeting sequence (Ogasawara et al., 2013) (Figure S9, S10). We tested whether the PTS1 signal peptide (*p‐35S*::*hph* – *p‐35Sx2*::*mVenus‐PTS1* construct) can be successfully used in *A. agrestis* Oxford for peroxisome targeting. We observed fluorescence in structures in the cytosol that resemble peroxisomes (Figure 8d).

The C‐terminus of mouse talin (mTalin) has been used for actin labelling in *A. thaliana* and *M. polymorpha* (Kimura & Kodama, 2016; Kost et al., 1998). We generated *A. agrestis* Oxford plants stably transformed with a *p‐35S*::*hph* – *p35S*::*mVenus‐mTalin* construct and we observed fluorescence localization in the filamentous structures in the cytosol (Figure 8e). To confirm that the observed structures are indeed actin filaments we treated thallus fragments with Latrunculin A (LatA), a reagent that depolymerizes actin. Actin filaments become disassembled after treatment with LatA indicating that mTalin can be used successfully in *A. agrestis* to target actin (Figure S11d,e).

The chloroplast transit peptide of the Rubisco small subunit (rbcS) has been used for protein targeting to the chloroplast in angiosperms (Kim et al., 2010; Shen et al., 2017). We cloned the predicted chloroplast transit peptide of the *A. agrestis* rbcS (Sc2ySwM_344.2836.1) (Figure S9). When fused with mTurquoise2 (*p‐AaEf1a*::*hph* – *AaEf1a*::*AarbcS‐mTurquoise2* construct) we observed fluorescence localization in the chloroplasts (Figure 8f).

In *M. polymorpha* the N‐terminal targeting sequence from a predicted chitinase (Mp2g24440) in combination with the C‐terminal HDEL ER retention peptide, has been successfully used for ER localization (Sauret‐Güeto et al., 2020). We tested the N‐terminal targeting sequence of a predicted chitinase in *A. agrestis* (Sc2ySwM_228.5627.1) (Figure S9), for its ability to direct fluorescent localization in ER (*p‐AaEf1a*::*hph* – *AaEf1a*::*mVenus‐AaChit* construct). We observed fluorescence in reticulate structures in the cytosol around the nuclei and in the plasma membrane (Figure 8g).

Finally, we tested the targeting sequence derived from the *A. thaliana Calcineurin B‐like 3* (*AtCBL3*) gene for tonoplast localization, which is functional in *M. polymorpha* (Figure S10). However, we were not able to detect any fluorescent signal in *A. agrestis* Oxford.

We also confirmed that the tags targeting mitochondria, Golgi, chloroplast, and ER can be also used for *A. punctatus*; however, the signal is not as strong as in *A. agrestis* (Figure S12). The number of lines obtained, and the constructs used for transformation for both *A. agrestis* and *A. punctatus* are given in Table 2.

**Table 2:** Summary of transgenic lines expressing different organelle targeting constructs

Construct	Species/strain	Number of transformants	Expression in rhizoids	Patchy expression	No fluorescence
*p‐AaEf1a*::*hph* – *AaEf1a*::*mVenus‐AaSD3*	Oxford	5	–	1	1
*p‐AaEf1a*::*hph* – *p‐35Sx2*::*mTurquoise2‐GmMan1*	Oxford	15	2		2
*p‐35S*::*hph* – *p‐35Sx2*::*mVenus‐PTS1*	Oxford	17	2	4	1
*p‐35S*::*hph* – *p‐35S*::*mVenus‐mTalin*	Oxford	5	–	–	–
*p‐AaEf1a*::*hph* – *AaEf1a*::*AarbcS‐mTurquoise2*	Oxford	14	2	–	–
*p‐AaEf1a*::*hph* – *AaEf1a*::*mVenus‐AaChit*	Oxford	5	–	–	1
*p‐AaEf1a*::*hph* – *p‐35Sx2*::*mVenus‐GLS‐ST*	Bonn	5	–	–	5
*p‐AaEf1a*::*hph* – *AaEf1a*::*mVenus‐AaSD3*	*A. punctatus*	5	–	–	1
*p‐AaEf1a*::*hph* – *p‐35Sx2*::*mTurquoise2‐GmMan1*	*A. punctatus*	5	–	–	1
*p‐AaEf1a*::*hph* – *AaEf1a*::*AarbcS‐mTurquoise2*	*A. punctatus*	6	–	–	1
*p‐AaEf1a*::*hph* – *AaEf1a*::*mVenus‐AaChit*	*A. punctatus*	5	–	–	1
*p‐AaEf1a*::*hph* – *p‐35S*::*mScarlet‐Lti6b*	Bonn	31	1	1	9

#### Applicability of the fluorescent protein mScarlet in hornworts

To expand the palette of fluorescent proteins (FP) that can be used in *A. agrestis*, we tested the expression of the synthetic monomeric Scarlet (mScarlet) ‘red’ fluorescent protein (Bindels et al., 2016). mScarlet is the brightest among the monomeric red FP with, for example, >3.5 times increase in brightness compared with mCherry. Given that its emission maximum is 594 nm, mScarlet can be combined with other FP such as eGFP, mVenus, or mTurquoise2 with minimal spectral overlap. We successfully generated *A. agrestis* Bonn lines expressing mScarlet (Figure 8h). The number of lines obtained are given in Table 2.

#### Applicability of the RUBY reporter and 2A peptides in hornworts

RUBY is a new reporter (He et al., 2020) that converts tyrosine to the red coloured betalain that is visible without the need for extra processing steps. Three betalain biosynthetic genes (*CYP76AD1*, *DODA*, and *Glucoysl transferase*) are fused into a single transcription unit using a single promoter the P2A self‐cleavage peptides and a terminator. We generated a construct where we fused the *AaTip1*;*1* promoter with the RUBY cassette and the double NOS‐35S terminator. However, when the plants transformed with the construct were examined, we did not observe any red colour (Figure S13a).

2A self‐peptides are known to have different cleavage efficiencies (Liu et al., 2017). To test the efficiency of 2A self‐cleavage peptides we generated two constructs where the mVenus FP fused to a nucleus targeting sequence and the mTurquoise2 FP fused to a plasma membrane targeting sequence were combined into a single transcription unit separated by either the P2A or the E2A peptide (Figure S13b–i). When used in *M. polymorpha* the expected FP localization was observed (Figure S13j,k). However, in the case of *A. agrestis* Oxford mainly mVenus localization into the nucleus was observed suggesting that cleavage efficiency is very low. The number of lines obtained are given in Table S2.

### New selection marker

The acetolactate synthase (ALS) is one of the enzymes that catalyses the biosynthesis of three essential amino acids, leucine, isoleucine, and valine. The herbicide chlorsulfuron is an ALS inhibitor and has been used in combination with mutated *ALS* genes as a selection marker in different plant species (Ogawa et al. 2008). We first tested whether *A. agrestis* is susceptible to chlorsulfuron and found that a 3‐week incubation with 0.5 μm chlorsulfuron was sufficient to inhibit growth of untransformed thallus tissue (Figure S14). We then performed trials where we used a *M*. *polymorpha* mutated *ALS* gene (Ishizaki et al., 2015) driven by the *AaEf1a* promoter as a selectable marker, the *GV3101 Agrobacterium* strain and 30 or 40 mm MES. Up to 18 chlorsulfuron resistant plants were successfully recovered from 200 mg of tissue (Figure 8i–k). It must be noted that growth of transgenic plants on chlorsulfuron selection media is slower compared with hygromycin. It takes approximately an additional 2 weeks until primary transformants are visible by naked eye.

## DISCUSSION

After testing several different approaches to increase transformation efficiency in the hornwort *A. agrestis*, it was pH control during co‐cultivation that proved most successful. Our results suggest that a pH of the co‐cultivation medium about 5.5 is a key parameter to increase *Agrobacterium* infection rates. A Similar observation has been for *A. thaliana Agrobacterium*‐mediated transformation (Wang et al., 2018). Consequently, our results support the notion of conserved innate immune responses via calcium signalling in land plants. These results may help to establish *Agrobacterium*‐mediated transformation methods for plants that currently lack an efficient transformation method (e.g., lycophytes or various streptophyte algal lineages).

## EXPERIMENTAL PROCEDURES

### Plant material and maintenance

In this study we used the *A. agrestis* Oxford and Bonn isolates (Szövényi et al., 2015) and *Anthoceros punctatus* (Li et al., 2014). Mature gametophytes and sporophytes of *A. punctatus* were originally collected from a glasshouse at the Royal Botanic Garden of Edinburgh, by Dr David Long. *Leiosporoceros dussii* was collected at Río Indio in Panama (N08°38.521′, W80°0.06.825, Elev. 801 m) by Juan Carlos Villarreal. *Phaeoceros carolinianus* collected in Louisiana, USA (provided by Fay‐Wei Li) and grown under similar axenic conditions with *A. agrestis*.

*A. agrestis*, *A. punctatus*, *L. dussii*, *and P. carolinianus* thallus tissue was propagated on KNOP medium (0.25 g L^−1^ KH_2_PO_4_, 0.25 g L^−1^ KCl, 0.25 g L^−1^ MgSO_4_•7H_2_O, 1 g L^−1^ Ca(NO_3_)_2_•4H_2_O, and 12.5 mg L^−1^ FeSO_4_•7H_2_O). The medium was adjusted to pH 5.8 with KOH and solidified using 7.5 g L^−1^ Gelzan CM (no. G1910; Sigma) in 92 × 16 mm Petri dishes (no. 82.1473.001; Sarstedt) with 25–30 ml of media per plate. Plants were routinely grown in a tissue culture room (21°C, 12 h of light and 12 h of dark, 3–5 or 35 μmol m^−2^ sec^−1^ light intensity; Philips TL‐D 58 W [835]). For the subculturing of the thallus tissue, a small part of it (approximately 2 mm × 2 mm) was cut using sterile disposable scalpels (no. 0501; Swann Morton) and placed on fresh media on a monthly basis (Figure S2).

*Marchantia polymorpha* accessions Cam‐1 (male) and Cam‐2 (female) were used in this study (Delmans et al., 2017). Plants were grown on half strength Gamborg B5 medium plus vitamins (Duchefa Biochemie G0210, pH 5.8) and 1.2% (w/v) agar (Melford capsules, A20021), under continuous light at 22°C with light intensity of 100 μmol m^−2^ sec^−1^.

### Tissue preparation for transformation

For the preparation of tissue used for transformation (fragmentation approach using razor blades), small pieces of thallus, approximately 2 mm × 2 mm, were cut using sterile disposable scalpels (no. 0501; Swann Morton) and placed on plates containing fresh growth medium (15–20 fragments per plate). The plates were grown for 4–6 weeks, at 21°C, 12 h of light, and 12 h of dark, 3–5 μmol m^−2^ sec^−1^ light intensity (Figure S2).

After 4–6 weeks, 1 g of thallus tissue (approximately 10 petri dishes) was transferred into an empty Petri dish, 2–10 ml of water was added and then the thallus fragmented using a razor blade (no. 11904325; Fisher Scientific) for approximately 5 min (Video S1). The fragmented tissue was washed with 100 ml of sterile water using a 100‐μm cell strainer (352 360; Corning) or until the flow through was clear (see Figures S5, S6, and S8).

When tissue was fragmented using a homogenizer, approximately 1 g of thallus tissue was homogenized in 20 ml sterile water using an Ultra‐Turrax T25 S7 Homogenizer (727407; IKA) and corresponding dispensing tools (10442743; IKA Dispersing Element), for 5 sec, using the lowest speed of 8000 rpm. The homogenized tissue was washed with 100 ml of sterile water using a 100‐μm cell strainer (352360; Corning) or until the flow through was clear.

### *Agrobacterium* culture preparation

One to three *Agrobacterium* colonies (*AGL1* strain) were inoculated in 5 ml of LB medium supplemented with rifampicin 15 μg ml^−1^ (no. R0146; Duchefa), carbenicillin 50 μg ml^−1^ (no. C0109; MELFORD) and the plasmid‐specific selection antibiotic spectinomycin 100 μg ml^−1^ (no. SB0901; Bio Basic). For the *GV3101* strain preparation, one to three colonies were inoculated in 5 ml of LB medium supplemented with rifampicin 50 μg ml^−1^ (no. R0146; Duchefa), gentamicin 25 μg ml^−1^ (no. G0124; Duchefa), and spectinomycin 100 μg ml^−1^ (no. SB0901; Bio Basic). The pre‐culture was incubated at 28°C for 2 days with shaking at 120 rpm.

### Co‐cultivation

Co‐cultivation medium was liquid KNOP with 1% (w/v) sucrose (0.25 g L^−1^ KH_2_PO_4_, 0.25 g L^−1^ KCl, 0.25 g L^−1^ MgSO_4_•7H_2_O, 1 g L^−1^ Ca(NO_3_)_2_•4H_2_O, 12.5 mg L^−1^ FeSO_4_•7H_2_O, and 10 g L^−1^ sucrose), supplemented with 40 mm MES, with pH 5.5 adjusted with KOH. The medium was filter sterilized (no. 430767; Corning Disposable Vacuum Filter/Storage Systems) and was stored in 50 ml Falcon tubes at −20°C.

Five millilitre of a 2‐day‐old *Agrobacterium* culture was centrifuged for 7 min at 2000 ***g***. The supernatant was discarded, and the pellet was resuspended in 5 ml liquid KNOP supplemented with 1% (w/v) sucrose, (S/8600/60; ThermoFisher, Loughborough, UK), 40 mm MES (255262A, ChemCruz), pH 5.5, and 100 μm 3′,5′‐dimethoxy‐4′‐hydroxyacetophenone (acetosyringone) (115540050; Acros Organics), dissolved in dimethyl sulfoxide (DMSO) (D8418; Sigma). The culture was incubated with shaking (120 rpm) at 28°C for 3–5 h. The fragmented thallus tissue was transferred into the wells of a six‐well plate (140675; ThermoFisher) with 5 ml of liquid KNOP medium supplemented with 1% (w/v) sucrose and 40 mm MES, and the pH was adjusted to 5.5. Then, 80 μl of *Agrobacterium* culture and acetosyringone at final concentration of 100 μm were added to the medium.

The tissue and *Agrobacterium* were co‐cultivated for 3 days with an orbital shaker at 110 rpm at 22°C without any additional supplementary light (only ambient light from the room, 1–3 μmol m^−2^ sec^−1^). After 3 days, the tissue was drained using a 100‐μm cell strainer (352360; Corning) and moved on to solid KNOP plates (two Petri dishes from a single well) supplemented with 100 μg ml^−1^ cefotaxime (BIC0111; Apollo Scientific, Bredbury, UK) and 10 μg ml^−1^ hygromycin (10687010; Invitrogen) or 0.5 μm chlorsulfuron (Sigma 64902‐72‐3; to prepare 1000× stock chlorsulfuron solution, 100 mm chlorsulfuron super‐stock solution was prepared in DMSO first, and then a 500 μm stock solution in dH_2_O).

If necessary, after 4 weeks, plants were transferred to fresh solid KNOP plates supplemented with 100 μg ml^−1^ cefotaxime and 10 μg ml^−1^ hygromycin and grown at 22°C under 12 h light: 12 h dark at a light intensity of 35 μmol m^−2^ sec^−1^ (TL‐D58W/835; Philips).

### Optimization trials

To test the effect of MES concentration on the transformation efficiency 3 g of *A. agrestis* Bonn thallus tissue was harvested 4 weeks after subculturing, fragmented with a razor blade, washed with 100 ml water, and distributed into six equal parts of 500 mg. Each part was then placed into a well of a six‐well plate with 5 ml co‐cultivation medium with different MES concentrations. The MES concentration of the co‐cultivation medium in each of the six wells was: 0, 10, 20, 30, 40, or 50 mm. One hundred microlitre of the same *Agrobacterium* pre‐culture, containing a *p‐AaEf1a*::*hph* – *p‐AaTip1*;*1‐eGFP‐Lti6b* construct, was added to each well. Three replicates of this trial were performed.

To test the effect of co‐cultivation medium pH value on the transformation efficiency, 2.5 g of *A. agrestis* Bonn thallus tissue was harvested 4 weeks after the subculture was made, fragmented with a razor blade, washed with 100 ml sterile distilled water, and distributed into five equal parts of 500 mg. Each part was then placed into a well of a six‐well plate with 5 ml co‐cultivation medium containing 40 mm MES, adjusted to different pH values. The pH value of the co‐cultivation medium in each of the five wells was adjusted with 1 m KOH to: 5, 5.25, 5.5, 5.75, or 6. One hundred microlitre of the same *Agrobacterium* pre‐culture, containing a *p‐AaEf1a*::*hph* – *p‐AaTip1*;*1‐eGFP‐Lti6b* construct, was added to each well. Three replicates of this trial were performed.

To test the effect of co‐cultivation sucrose concentration on the transformation efficiency, approximately 1.2 g of *A. agrestis* Bonn thallus tissue was harvested 4 weeks after subculturing, fragmented with a razor blade, washed with 100 ml water, distributed into six equal parts of 200 mg, and each part was then placed into a well of a six‐well plate. In each plate, the sucrose concentration in three wells was 1% (w/v) and in the remaining three was 2% (w/v). Eighty microlitre of the same *Agrobacterium* (transformed with the *p‐AaEf1a*::*hph* – *35S_s*::*eGFP‐Lti6b* construct) pre‐culture was added to each well. The experimental set‐up was similar for testing the effect of the *Agrobacterium* strain (*AGL1* or *GV3101*) on the transformation efficiency as well as the *A. agrestis* Oxford optimization trials with the only difference that the *Agrobacterium* was transformed with the *p‐AaEf1a*::*hph* – *p‐AaEf1a*::*eGFP‐Lti6b* construct.

Co‐cultivation and selection were carried out as described in the previous section. After 3–4 weeks on a first round of selection, surviving and growing thallus fragments were transferred to fresh selection plates. After an additional 2 months on second selection, growing thallus pieces that showed GFP expression (observed with a Leica M205 FA Stereomicroscope, GFP longpass filter) were counted as stable transformants.

### *Marchantia polymorpha* transformation

Transgenic *M. polymorpha* plants were obtained according to Sauret‐Güeto et al. (2020).

### Construct generation

Constructs were generated using the OpenPlant toolkit (Sauret‐Güeto et al., 2020). OpenPlant L0 parts used: OP‐019 CDS_mALS, OP‐023 CDS12‐eGFP, OP‐020 CDS_hph, OP‐027 CDS12_mTurquoise2, OP‐029 CDS12_mVenus, OP‐037 CTAG_Lti6b, OP‐054 3TER, _Nos‐35S and OP‐049 PROM_35S (for detailed maps see Table S1). The DNA sequence of target peptides described in this study were synthesized using the Genewiz or the IDT company and cloned into the pUAP4 vector. The information about the sequences of the target peptides used in this study can be found in Figure S9.

### Western blotting

Fifty milligram of *A. agrestis* tissue, grown for 3 weeks on KNOP medium at 21°C in 12 h light (5 μmol m^−2^ sec^−1^)/12 h dark, were placed in 1.5 ml tube with two metal beads, flash frozen in liquid nitrogen and grounded using a TissueLyser II (no. 85300; Qiagen) at 30 Hz for 5 min. The tissue powder was resuspended in 400 μl 10× Laemmli loading buffer (0.5 m Tris–HCl pH 6.8, 20% w/v sodium dodecyl sulphate, 30% v/v glycerol, 1 m dithiothreitol, 0.05% w/v bromophenol blue,) supplemented with Roche cOmplete Protease Inhibitor (no. 11836170001; Roche), heated at 90°C for 10 min and centrifuged at 10 000 ***g*** for 5 min. The supernatant was transferred to a new tube. Equal amounts of proteins were separated by denaturing electrophoresis in NuPAGE gel (no. NP0322BOX; Invitrogen) and electrotransferred to nitrocellulose membranes using the iBlot2 Dry Blotting System (ThermoFisher). eGFP was immune‐detected with anti‐GFP antibody (1:4000 dilution) (JL‐8, no. 632380; Takara) and antimouse horseradish peroxidase (1:15 000 dilution) (no. A9044; Sigma) antibodies. Actin was immune‐detected with anti‐actin (plant) (1:1500 dilution) (no. A0480; Sigma) and (1:15 000 dilution) antimouse horseradish peroxidase (1:15 000 dilution) (no. A9044, Sigma) antibodies, using the iBind™ Western Starter Kit (no. SLF1000S; ThermoFisher). Western blots were visualized using the ECL™ Select Western Blotting Detection Reagent (no. GERPN2235, GE) following the manufacturer's instructions. Images were acquired using a Syngene Gel Documentation system G:BOX F3.

### Sample preparation for imaging

A gene frame (no. AB0576; ThermoFisher) was positioned on a glass slide. A thallus fragment was placed within the medium‐filled gene frame together with 30 μl of MilliQ water. The frame was then sealed with a coverslip. Plants were imaged immediately using a Leica SP8X spectral fluorescent confocal microscope.

### Imaging with confocal microscopy

Images were acquired on a Leica SP8X spectral confocal microscope. Imaging was conducted using either a 10× air objective (HC PL APO 10×/0.40 CS2) or a 20× air objective (HC PL APO 20×/0.75 CS2). Excitation laser wavelength and captured emitted fluorescence wavelength windows were as follows: for mTurquoise2 (442 nm, 460–485 nm), for eGFP (488 nm, 498–516 nm), for mVenus (515 nm, 522–540 nm), and for chlorophyll autofluorescence (488 or 515, 670–700 nm).

Sporophytes of promoter‐eGFP reporter lines were dissected from the gametophyte, and the gametophyte tissue around the base carefully removed with scalpels under a dissecting scope without disturbing the sporophyte base. The sporophytes were then transferred into a Lab‐Tek chambered coverglass (no. 155361; Lab‐Tek) and overlaid with 5 mm solid KNOP medium (solidified with Gelrite) and water to keep them in place and prevent desiccation. eGFP expression within the sporophyte base of the living sporophytes was then visualized with a Leica TCS SP8 MP, excitation with a multiphoton laser at 976 nm, eGFP fluorescence detected with a bandpass filter (HyD‐RLD 2ch FITC filter 525/50), and chloroplast autofluorescence with HyD‐RLD 675/55.

### Light microscopy

Images were captured using a KEYENCE VHX‐S550E microscope (VHX‐J20T lens) or a Leica M205 FA Stereomicroscope (with GFP longpass filter).

### MitoTracker staining

A 1 mm MitoTracker Red CMXRos (no. 9082; Cell Signaling Technology, UK) stock solution was prepared in DMSO. Stock solution was diluted 100 times in distilled water and added to small *A. agrestis* thallus fragments (approximately 2 × 2 mm) and then incubated at room temperature for 1 h. Plant fragments (*n* = 5) were washed with distilled water four times and then imaged immediately using a confocal microscope. Excitation laser wavelength and captured emitted fluorescence wavelength window were as follows: 579 nm, 590–610 nm.

### Latrunculin a treatment

A stock solution of 0.1 mm Latrunculin A (no. 9082; Abcam) in DMSO was prepared. For the LatA treatment, the stock solution was diluted 10 times in distilled water. For the control, DMSO was diluted 10 times in distilled water. Two gene frames (no. AB0576; ThermoFisher) were positioned on top of each other on a glass slide. A thallus fragment was placed within the gene frames together with approximately 60 μl of diluted LatA or diluted DMSO. Plants were imaged immediately using a Leica SP8X spectral fluorescent confocal microscope. Three independent experiments were performed.

## AUTHOR CONTRIBUTIONS

MW, EF, and PS conceived and designed the experiments. EF and MW performed optimizations, and generated and characterized the hornwort transgenic lines. EF, MW, and AOM performed imaging. KSR collected, processed and imaged hornwort specimens in the light and transmission electron microscope. JR and SSG generated and characterized the transgenic *M. polymorpha* lines. JH and JMH provided resources. CRRS performed *A. punctatus* optimizations. MW, EF, and PS wrote the article with contributions from all the authors.

## CONFLICT OF INTEREST

The authors declare that they have no conflicts of interest associated with this work.

## Supporting information


**Video S1.** Fragmenting *A. agrestis* thallus using a razor blade.

**Figure S1.** Sporophyte induction for *A. agrestis* Bonn.
**Figure S2.** Tissue culturing for *A. agrestis*, *A. punctatus*, *L. dussii*, and *P. carolinianus*.
**Figure S3.** Effect of different MES concentrations in the transformation buffer on *A. agrestis* Bonn tissue fragments after 3 days of co‐cultivation.
**Figure S4.** Activity of the *AaTip1*;*1* and the *AaE1a* promoters in the gametophyte of *A. agrestis*.
**Figure S5.** Workflow showing steps of the transformation protocol optimized for the *A. agrestis* Bonn strain.
**Figure S6.** Workflow showing steps of the transformation protocol optimized for the *A. agrestis* Oxford strain.
**Figure S7.** Workflow showing steps of the protocol used to transform *A. punctatus*.
**Figure S8.** Workflow showing steps of the protocol used to transform *L. dussii*.
**Figure S9.** Summary of sequences used as transit peptides in this study to tailor specific localization of fluorescent proteins.
**Figure S10.** Localization of fluorescent proteins tagged with various transit peptides in the liverwort *Marchantia polymorpha*.
**Figure S11.** MitoTracker and LatA treatment.
**Figure S12.** Targeting fluorescent proteins to various subcellular compartments in the hornwort *A. punctatus*.
**Figure S13.** Testing utility of the RUBY reporter and the 2A self cleavage peptides in *A. agrestis*.
**Figure S14.** Chlorsulfuron sensitivity of *A. agrestis* gametophytes.
**Table S1.** Plasmid construct sequences.
**Table S2.** Number and expression of RUBY constructs.
