# Supplementary figures and images for: An optimized transformation protocol for Anthoceros agrestis and three more hornwort species
Source: Plant J. 2023 Apr 11;114(3):699–718. doi: 10.1111/tpj.16161 (PMC10952725; doi:10.1111/tpj.16161)

## Slide 1
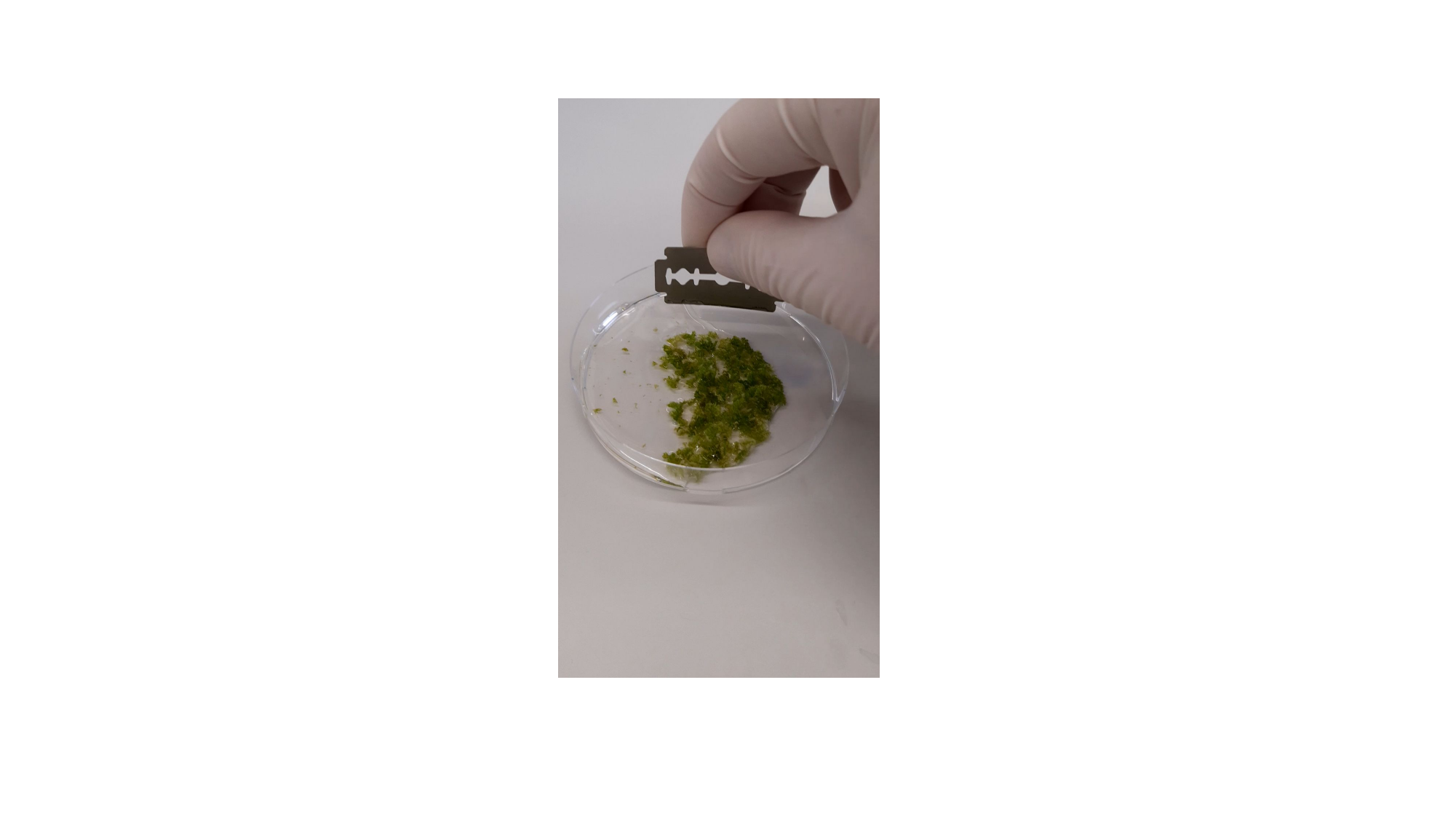

Supplement: Supplementary file 1 — Video S1. Fragmenting A. agrestis thallus using a razor blade. [file TPJ-114-699-s002.pptx]
