# Supplementary material for: An optimized transformation protocol for Anthoceros agrestis and three more hornwort species
Source: Plant J. 2023 Apr 11;114(3):699–718. doi: 10.1111/tpj.16161 (PMC10952725; doi:10.1111/tpj.16161)
Supplement: Supplementary file 2 — Figure S1. Sporophyte induction for A. agrestis Bonn. Figure S2. Tissue culturing for A. agrestis, A. punctatus, L. dussii, and P. carolinianus. Figure S3. Effect of different MES concentrations in the transformation buffer on A. agrestis Bonn tissue fragments after 3 days of co‐cultivation. Figure S4. Activity of the AaTip1;1 and the AaE1a promoters in the gametophyte of A. agrestis. Figure S5. Workflow showing steps of the transformation protocol optimized for the A. agrestis Bonn strain. Figure S6. Workflow showing steps of the transformation protocol optimized for the A. agrestis Oxford strain. Figure S7. Workflow showing steps of the protocol used to transform A. punctatus. Figure S8. Workflow showing steps of the protocol used to transform L. dussii. Figure S9. Summary of sequences used as transit peptides in this study to tailor specific localization of fluorescent proteins. Figure S10. Localization of fluorescent proteins tagged with various transit peptides in the liverwort Marchantia polymorpha. Figure S11. MitoTracker and LatA treatment. Figure S12. Targeting fluorescent proteins to various subcellular compartments in the hornwort A. punctatus. Figure S13. Testing utility of the RUBY reporter and the 2A self cleavage peptides in A. agrestis. Figure S14. Chlorsulfuron sensitivity of A. agrestis gametophytes. Table S1. Plasmid construct sequences. Table S2. Number and expression of RUBY constructs. [file TPJ-114-699-s001.docx]

**Supplemental Information**

**An optimised transformation protocol for *Anthoceros agrestis* and three more hornwort species.**

Manuel Waller^1,2^*, Eftychios Frangedakis^3,^*^,$^, Alan Marron^3^, Susanna Sauret- Güeto^3,4^, Jenna Rever^3^,Cyrus Raja Rubenstein Sabbagh^5^**,** Julian M. Hibberd^3^, Jim Haseloff^3^, Karen Renzaglia^6^, and Péter Szövényi^1,2,$^

^1^Department of Systematic and Evolutionary Botany, University of Zurich, Switzerland

^2^Zurich-Basel Plant Science Center, Zurich, Switzerland

^3^Department of Plant Sciences, University of Cambridge, Cambridge, CB3 EA, UK

^4^Present address: Crop Science Centre, University of Cambridge, 93 Lawrence Weaver Road, Cambridge CB3 0LE, UK

^5^Department of Microbiology and Molecular Genetics, College of Biological Sciences, University of California, Davis, California 95616, USA

^6^Department of Plant Biology, Southern Illinois University, Carbondale, IL 62901, USA

* equal contribution

$ correspondence:

Péter Szövényi

Eftychios Frangedakis


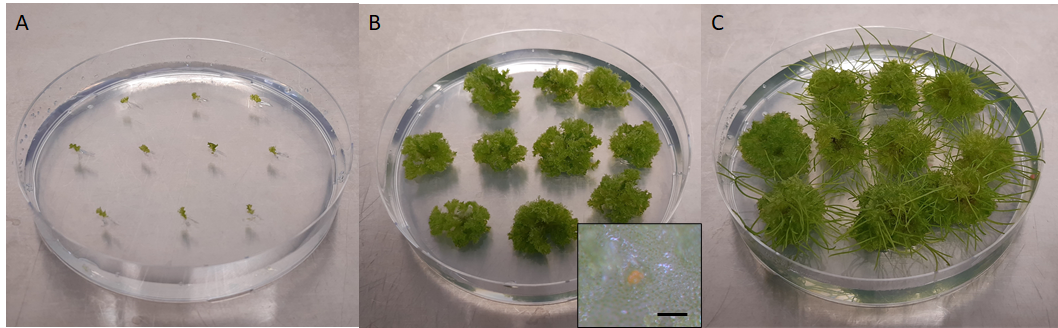


**Figure S1: Sporophyte induction for *A. agrestis* Bonn**

To induce development of sporophytes, *A. agrestis* Bonn thallus fragments were sub-cultured as described in Methods, under Plant material and maintenance (A). The established plant cultures were then kept in a growth chamber at 24h light, 20-30 μmol m^−2^ s^−1^, 60% humidity, 23°C and grown for approx. 1.5 - 2 months, until the individual thallus pieces would grow into clumps of tissue with a diameter of approximately 1-2 cm (B). At this point, the cultures were examined under the stereomicroscope and if antheridia could be observed (B), approx. 5 ml of sterile water was poured over the tissue and added to the plate. The plates were then incubated for another 2-3 weeks in the growth chamber, after which the appearance and growth of sporophytes could be observed (C) (scale bar: 100 μm).


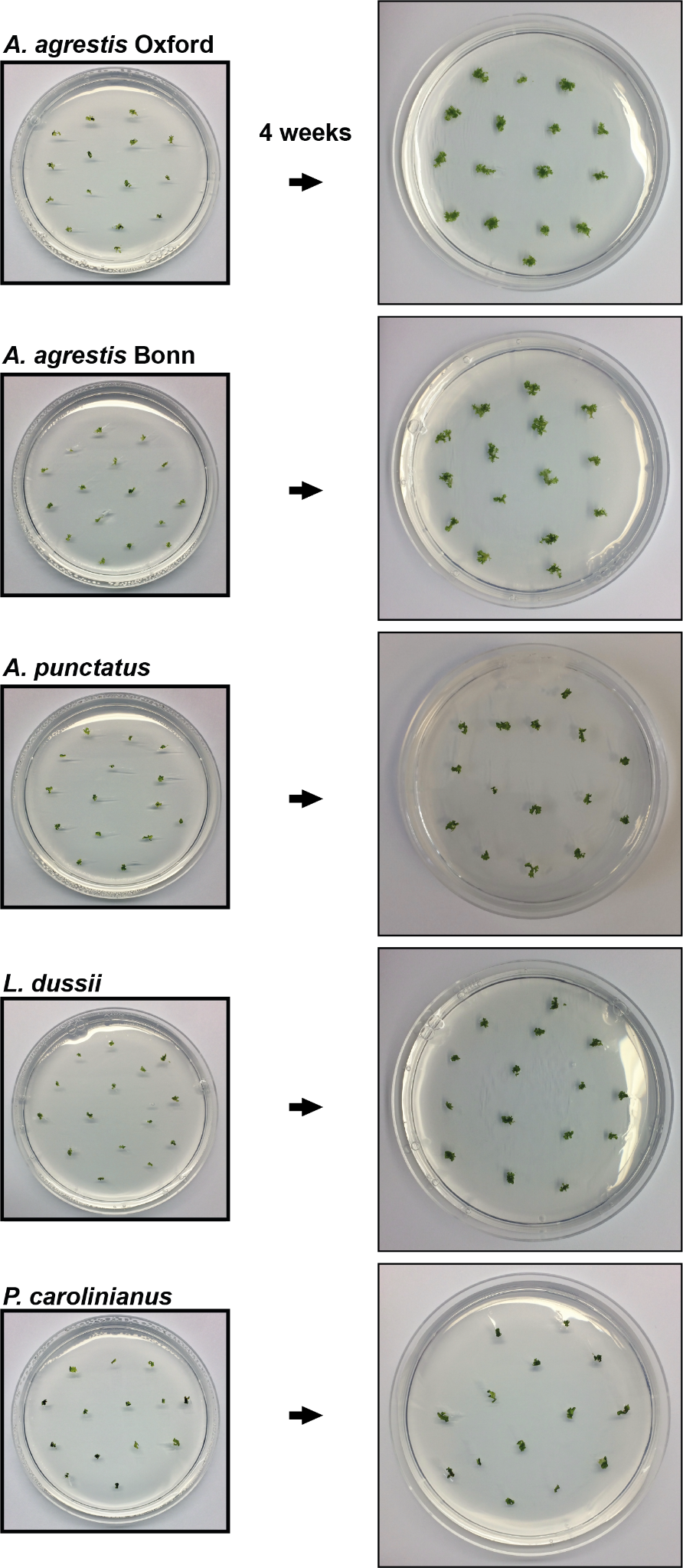


**Figure S2: Tissue culturing for *A. agrestis, A. punctatus, L. dussii* and *P. carolinianus.***

Images to demonstrate morphology of thallus used for routine tissue propagation for *A. agrestis, A. punctatus, L. dussii* and *P. carolinianus.*

Petri dish dimensions: 92 x16 mm.


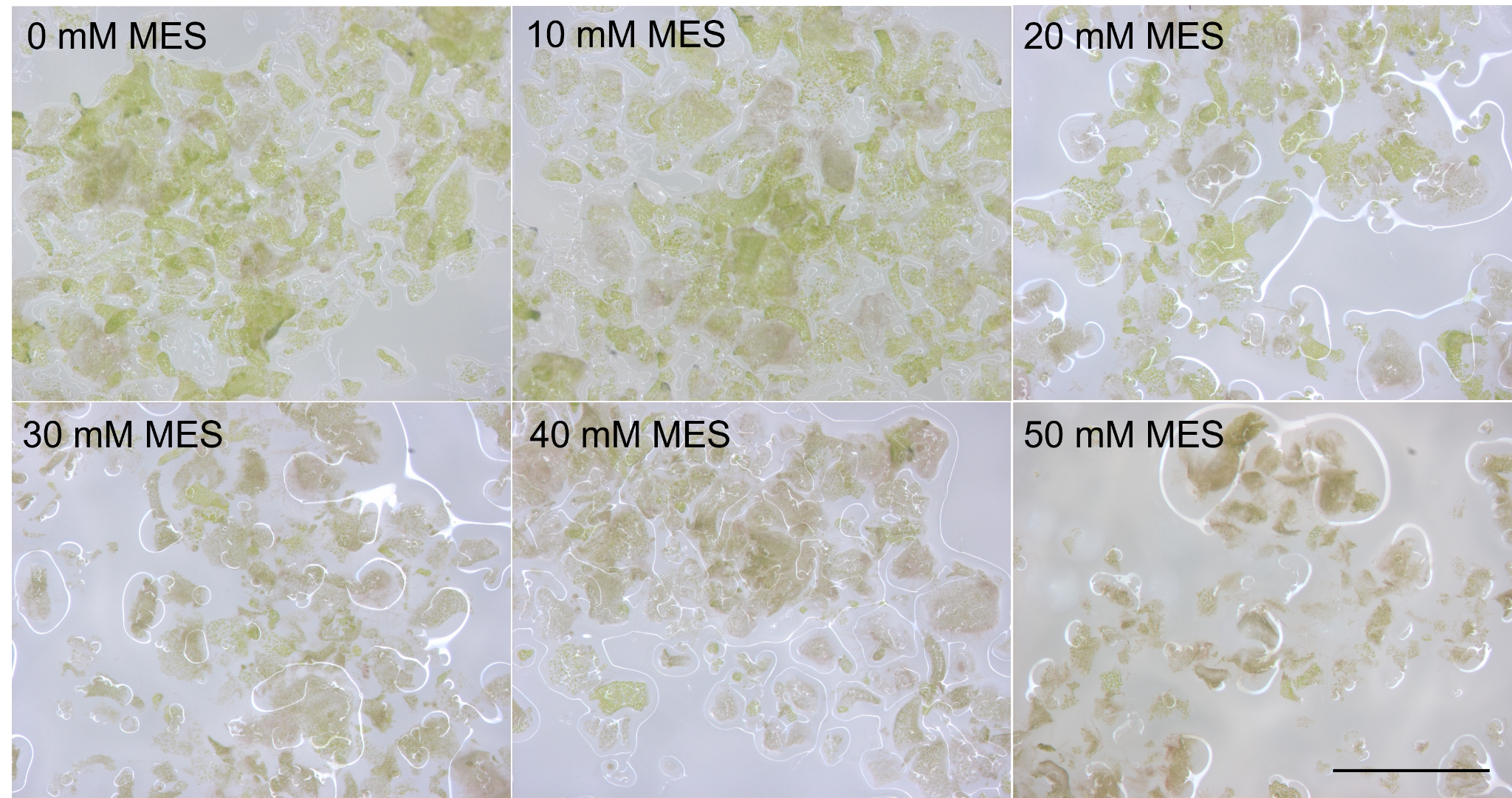


**Figure S3: Effect of different MES concentrations in the transformation buffer on *A. agrestis* Bonn tissue fragments after 3 days of co-cultivation.**

With increased MES concentration, tissue fragments look unhealthy or dead 3 days after co-cultivation as indicated by the brown/grey colour of the chloroplasts. At 50 mM MES, almost no living cells (with green chloroplasts) could be found (scale bar : 1 mm).


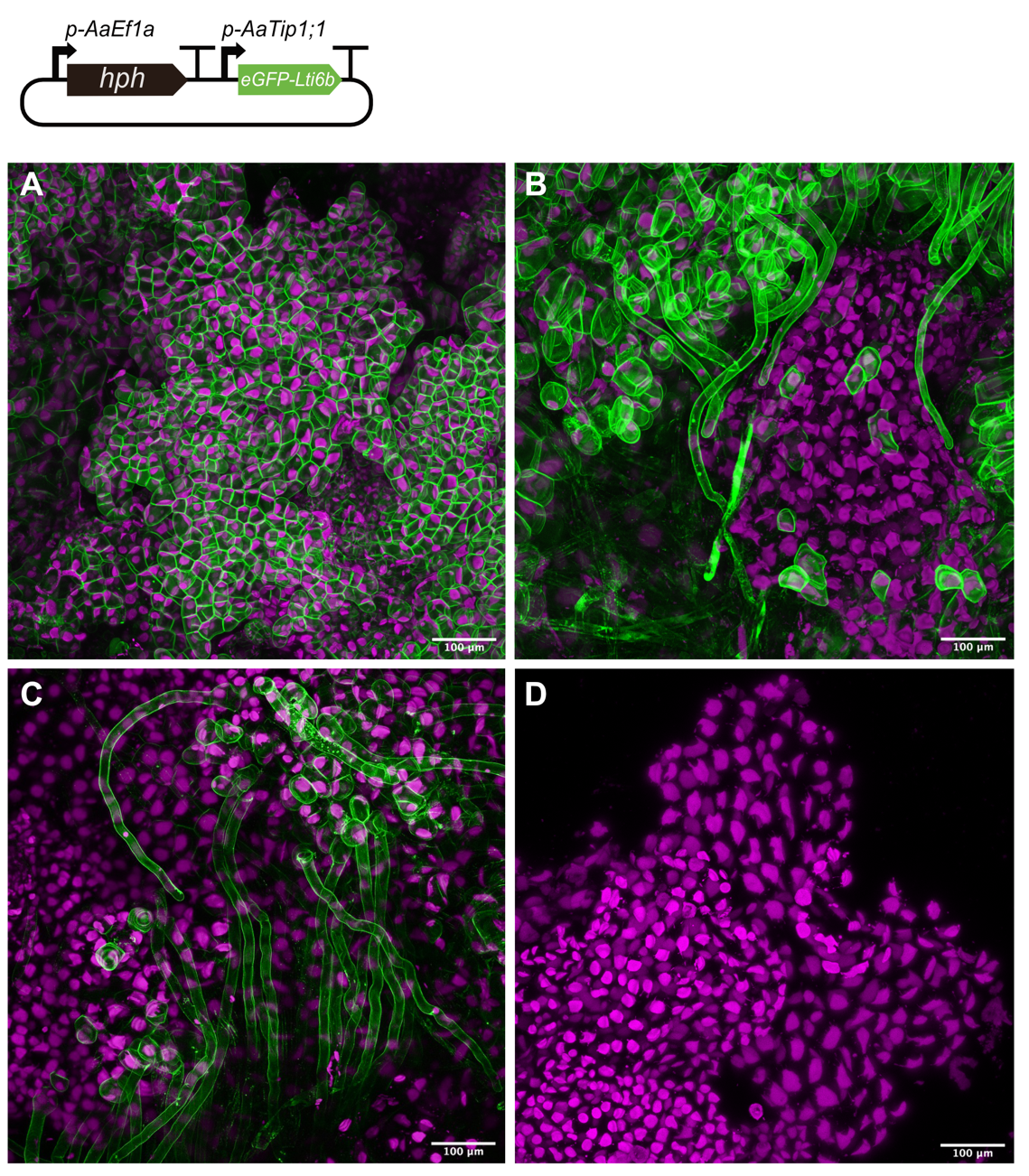


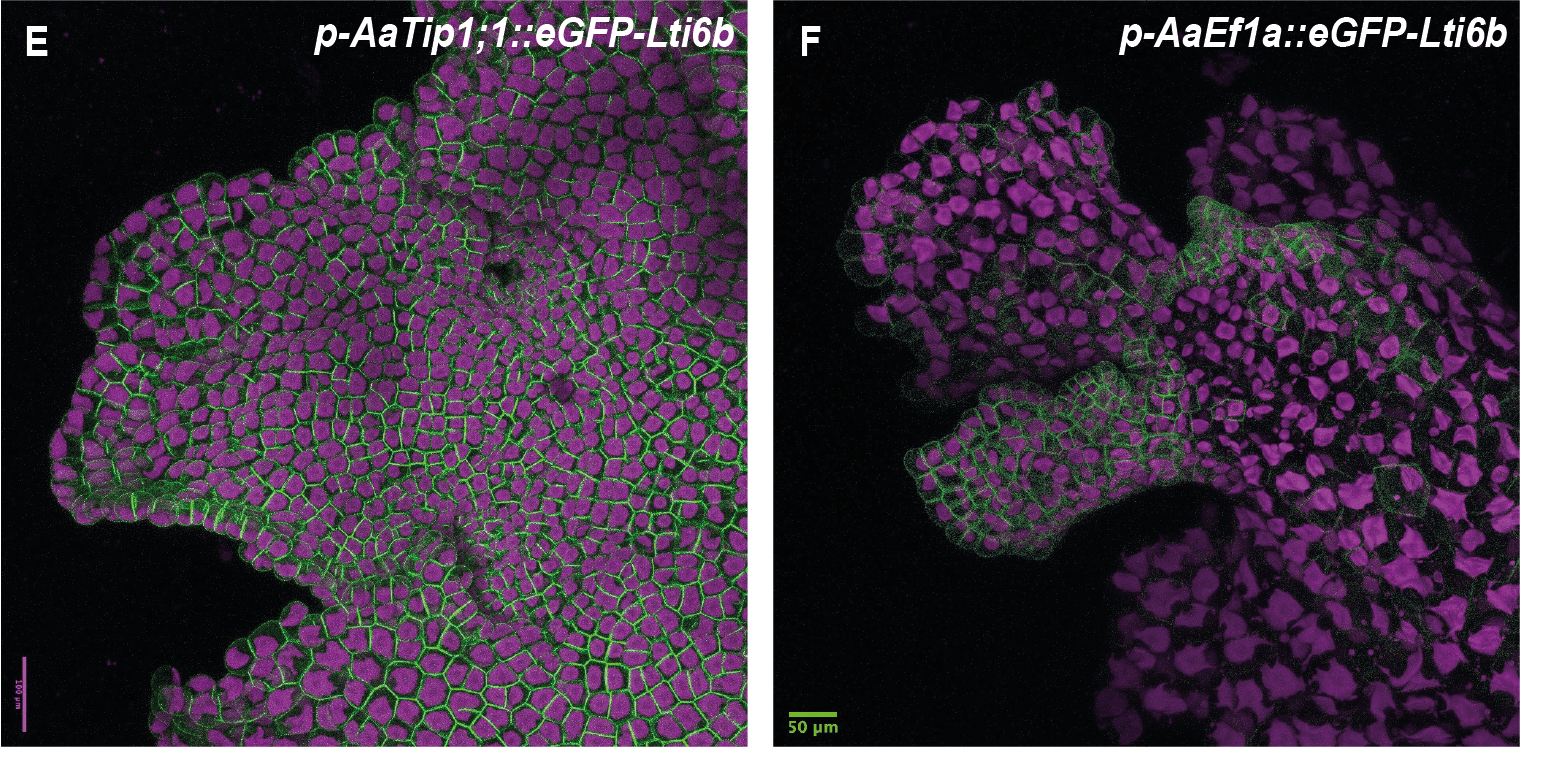


**Figure S4: Activity of the *AaTip1;1* and the *AaEf1a* promoters in the gametophyte of *A. agrestis*.**

On the top of the figure: Schematic representation of the construct for the expression of two transcription units (TU): one TU for the expression of the hygromycin B *phosphotransferase* (*hph*) gene under the control of the *AaEf1a* promoter and one TU for the expression of *p-AaTip1;1::eGFP-Lti6b.* A-D) Confocal images of *A. agrestis* Bonn transformed with the *p-AaTip1;1::eGFP-Lti6b* construct. A) Thallus margin. B) Rhizoids and irregular thallus tissue expression. C) Rhizoid only expression. D) Section of the thallus not expressing eGFP. Scale bars: 100 μm. E) Gametophyte thallus margin of *A. punctatus* transformed with the *p-AaEf1a::hph - p-AaTip1;1::eGFP-Lti6b* construct. Scale bar: 100 μm. F) Gametophyte thallus margin of *A. punctatus* transformed with the *p-AaEf1a::hph - p*-*AaEf1a::eGFP-Lti6b* construct. Scale bar: 50 μm. All construct maps at Supp Table 1.


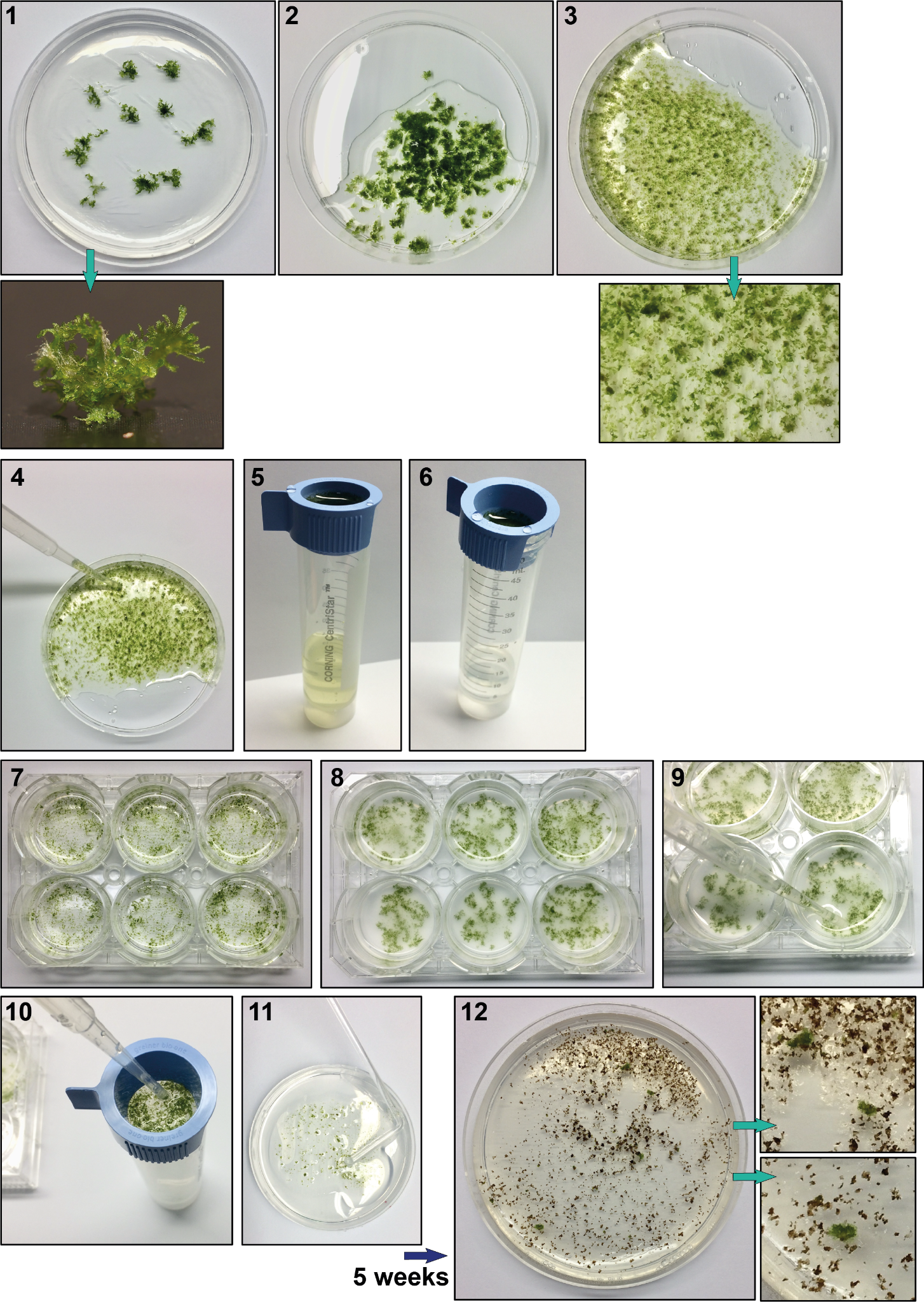


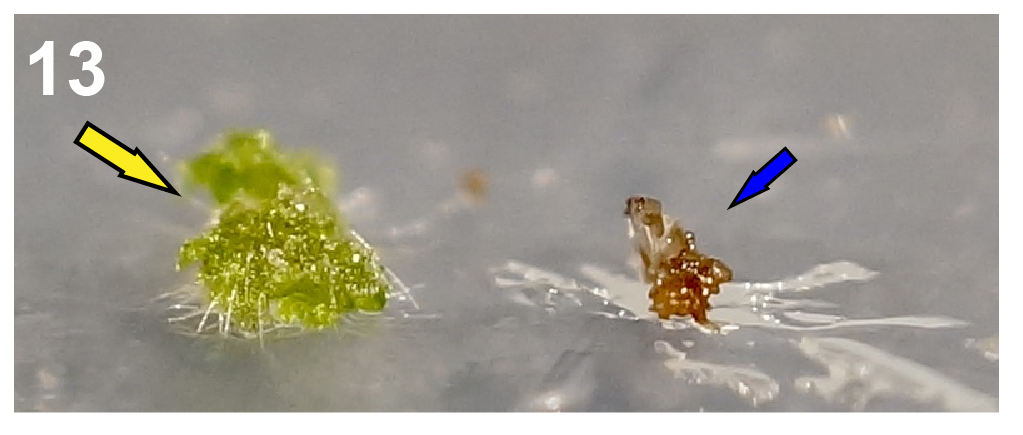


**14**


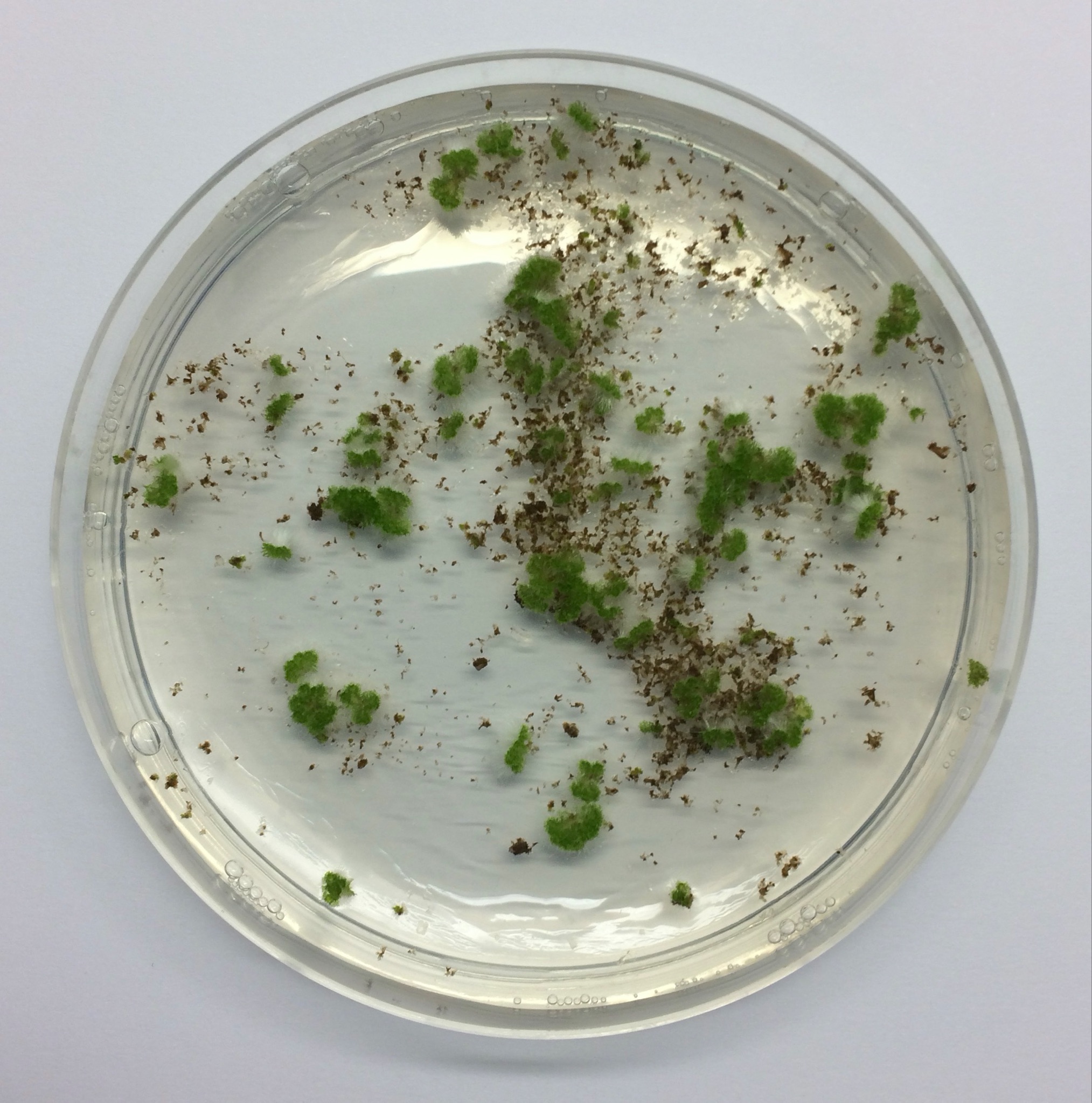


**Figure S5: Workflow showing steps of the transformation protocol optimised for the *A. agrestis* Bonn strain.**

**1)** Approximately 1 g of thallus tissue grown for 4 weeks under low light intensity was collected (approximately 0.1 g of tissue per petri dish - 10 petri dishes in total). **2)** Tissue was transferred into an empty petri dish, sterile water was added until the tissue was covered **3)** the tissue was fragmented using a razor blade (for 5 mins in total). **4)** the tissue was transferred from the petri dish into a cell strainer positioned on a falcon tube using a plastic pipette. **5-6)** the tissue was washed using ~100 ml of sterile water or until the flow through was clear. **7)** The fragmented thallus tissue was transferred into a 6-well plate (transfer 1⁄6 of the 1 g tissue into a single well) with 5 ml of liquid KNOP medium supplemented with 1% (w/v) sucrose and 40 mM MES, 80 μL of *Agrobacterium* culture and acetosyringone at final concentration of 100 μM. **8)** The tissue was co-cultivated with the *Agrobacterium* for 3 days on a shaker at 110 rpm, with only ambient light. **9-10)** Using a sterile plastic pipette the tissue of one well was transferred into a cell strainer, drained and then transferred on growth media containing the appropriate antibiotic (onto 1 petri dish from one well). **11)** To facilitate spreading of the tissue, 2 ml of sterile water was added to the petri dish. **12)** After 4-6 weeks successful transformants were visible on the petri dish (successful transformants can be identified using a dissecting scope after 4 weeks selection based on rhizoid production and/or fluorescence if such a marker is present on the construct). **13)** The emergence of rhizoids is an indication of successful transformation (yellow arrow: transformed thallus fragment, blue arrow: dying thallus fragment). To eliminate false positives, surviving tissue fragments were transferred again on antibiotics containing growth media. **14)** Example of plate with successful transformants 8 weeks after co-cultivation.

Petri dish dimensions: 92 x16 mm.

**
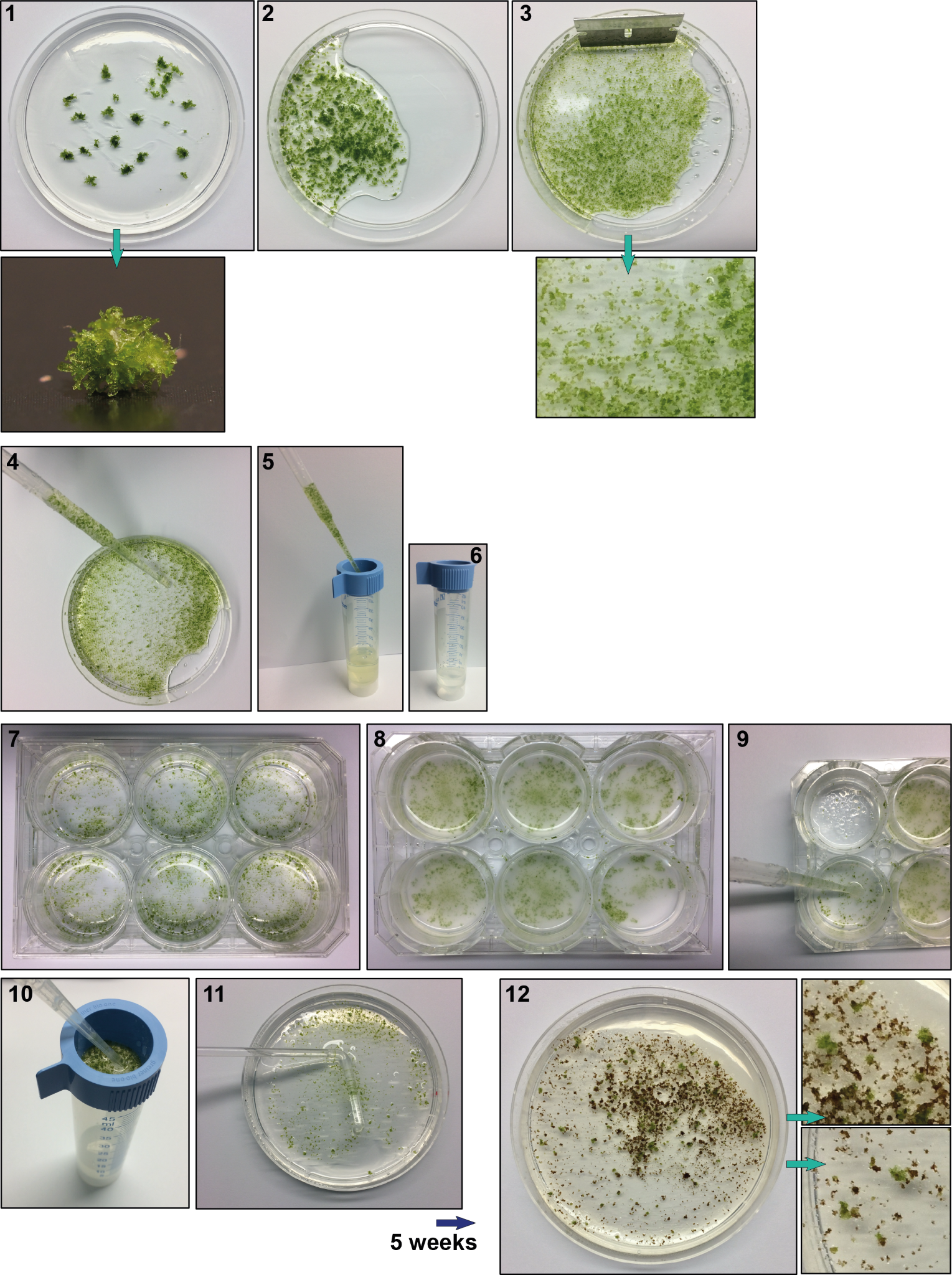
**

**
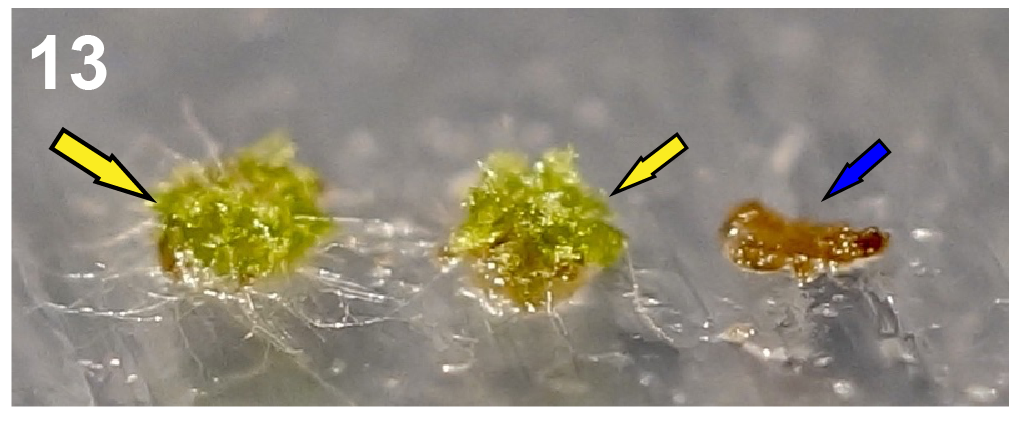
**

**14**


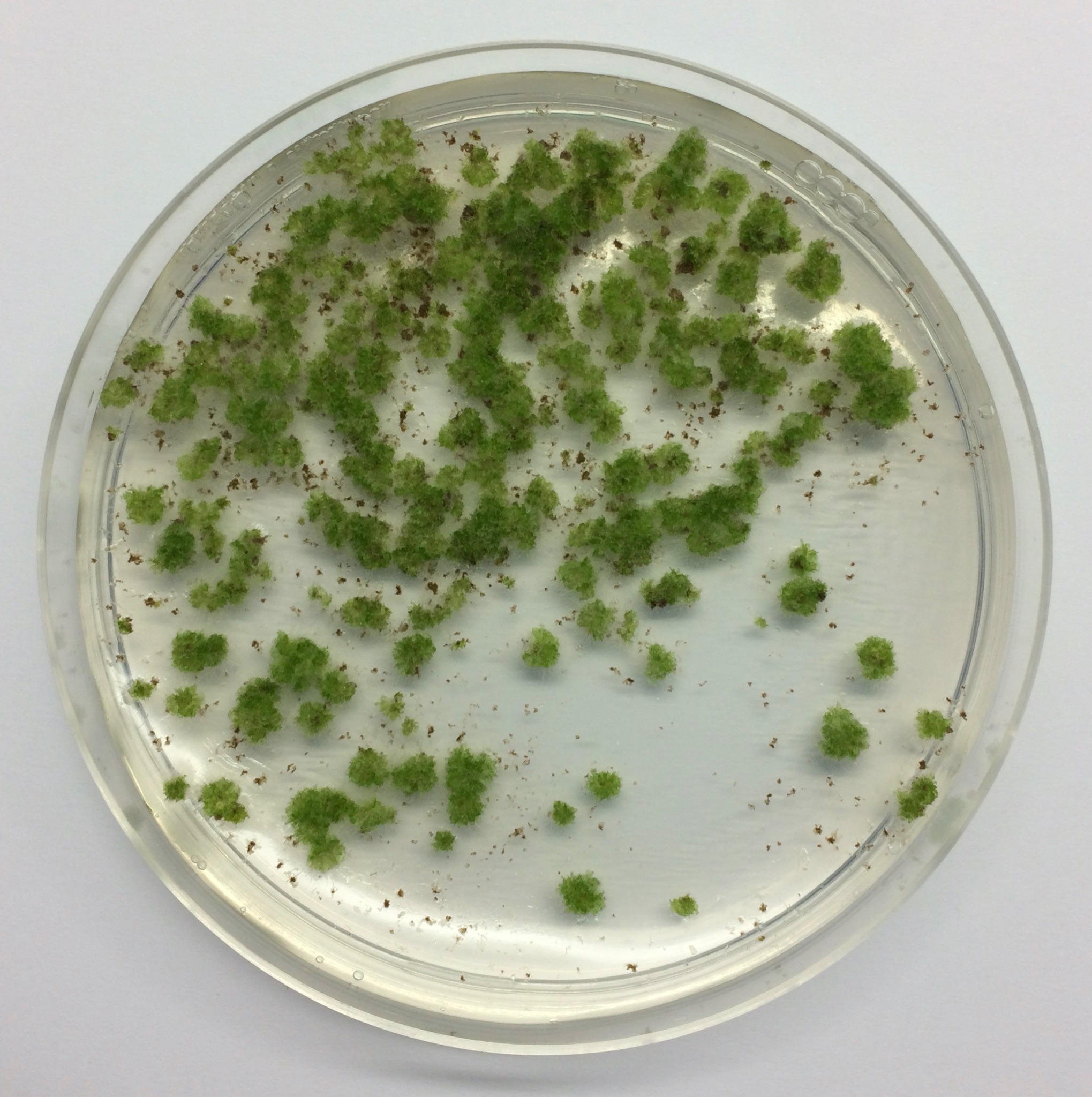


**Figure S6:** **Workflow showing steps of the transformation protocol optimised for the *A. agrestis* Oxford strain.**

**1)** Approximately 1 g of thallus tissue grown for 4 weeks under low light intensity was collected (approximately 0.1 g of tissue per petri dish - 10 petri dishes in total). **2)** Tissue was transferred into an empty petri dish, sterile water was added until the tissue was covered **3)** the tissue was fragmented using a razor blade (for 5 mins). **4)** the tissue was transferred from the petri dish into a cell strainer positioned on a falcon tube using a plastic pipette. **5-6)** the tissue was washed using ~100 ml of sterile water or until the flow through was clear. **7)** The fragmented thallus tissue was transferred into a 6-well plate (transfer 1⁄6 of the 1 g tissue into a single well) with 5 ml of liquid KNOP medium supplemented with 1% (w/v) sucrose and 40 mM MES, 80 μl of *Agrobacterium* culture and acetosyringone at final concentration of 100 μM. **8)** The tissue was co-cultivated with the *Agrobacterium* for 3 days on a shaker at 110 rpm, with only ambient light. **9-10)** Using a sterile plastic pipette the tissue of one well was transferred into a cell strainer, drained and then transferred on growth media containing the appropriate antibiotic (onto 1 petri dish from one well). **11)** To facilitate spreading of the tissue, 2 ml of sterile water was added to the petri dish. **12)** After 4-6 weeks successful transformants were visible on the petri dish (successful transformants can be identified using a dissecting scope after 4 weeks selection based on rhizoid production and/or fluorescence if such a marker is present on the construct). **13)** The emergence of rhizoids is an indication of successful transformation (yellow arrows: transformed thallus fragment, blue arrow: dying thallus fragment). To eliminate false positives, surviving tissue fragments were transferred again on antibiotics containing growth media. **14)** Example of plate with successful transformants 8 weeks after co-cultivation.

Petri dish dimensions: 92 x16 mm.


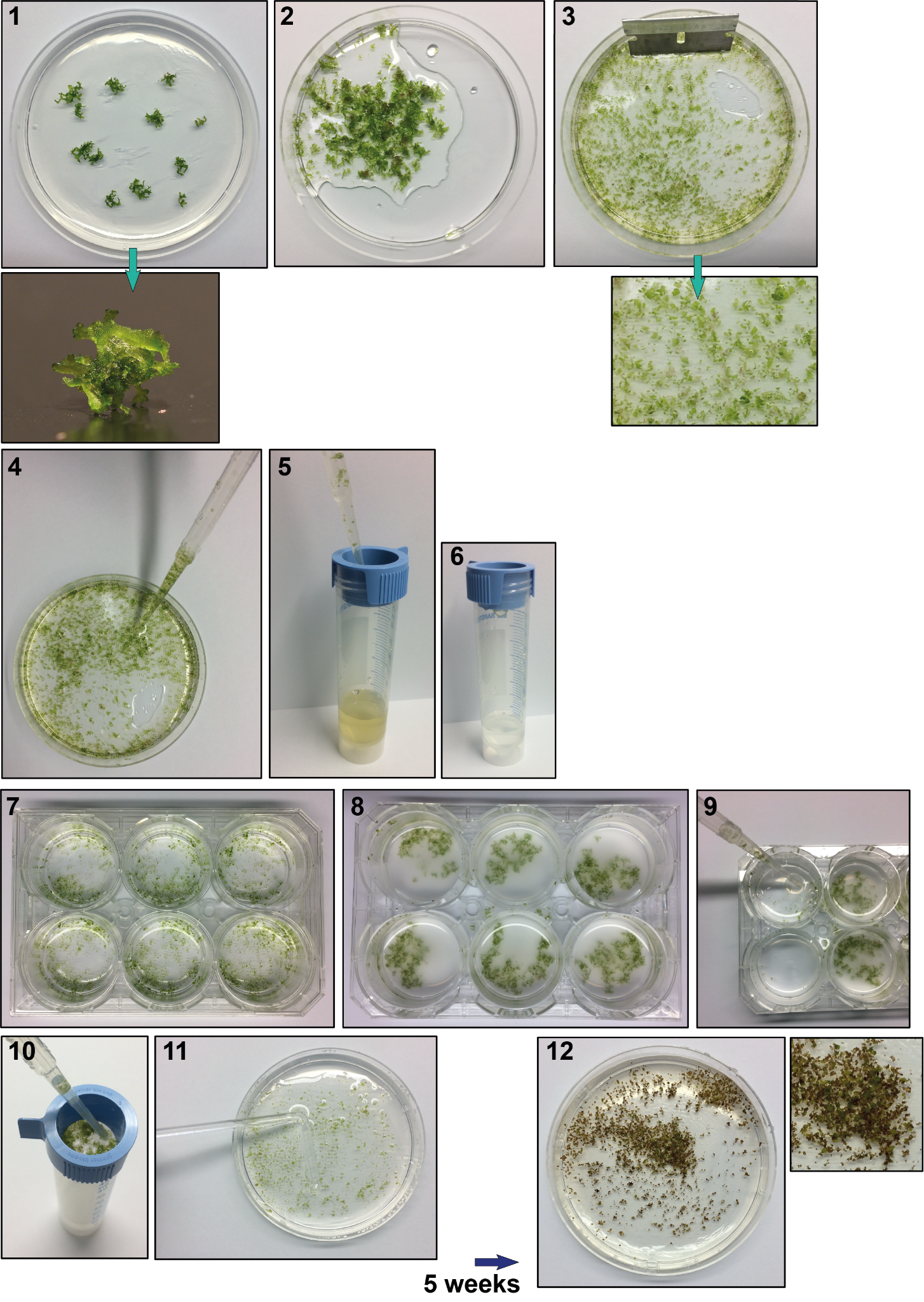


**
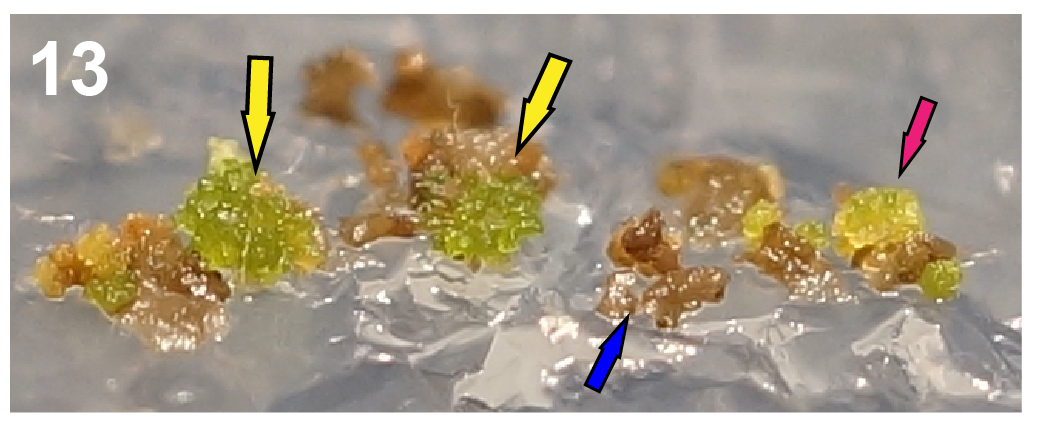
**

**14**

**
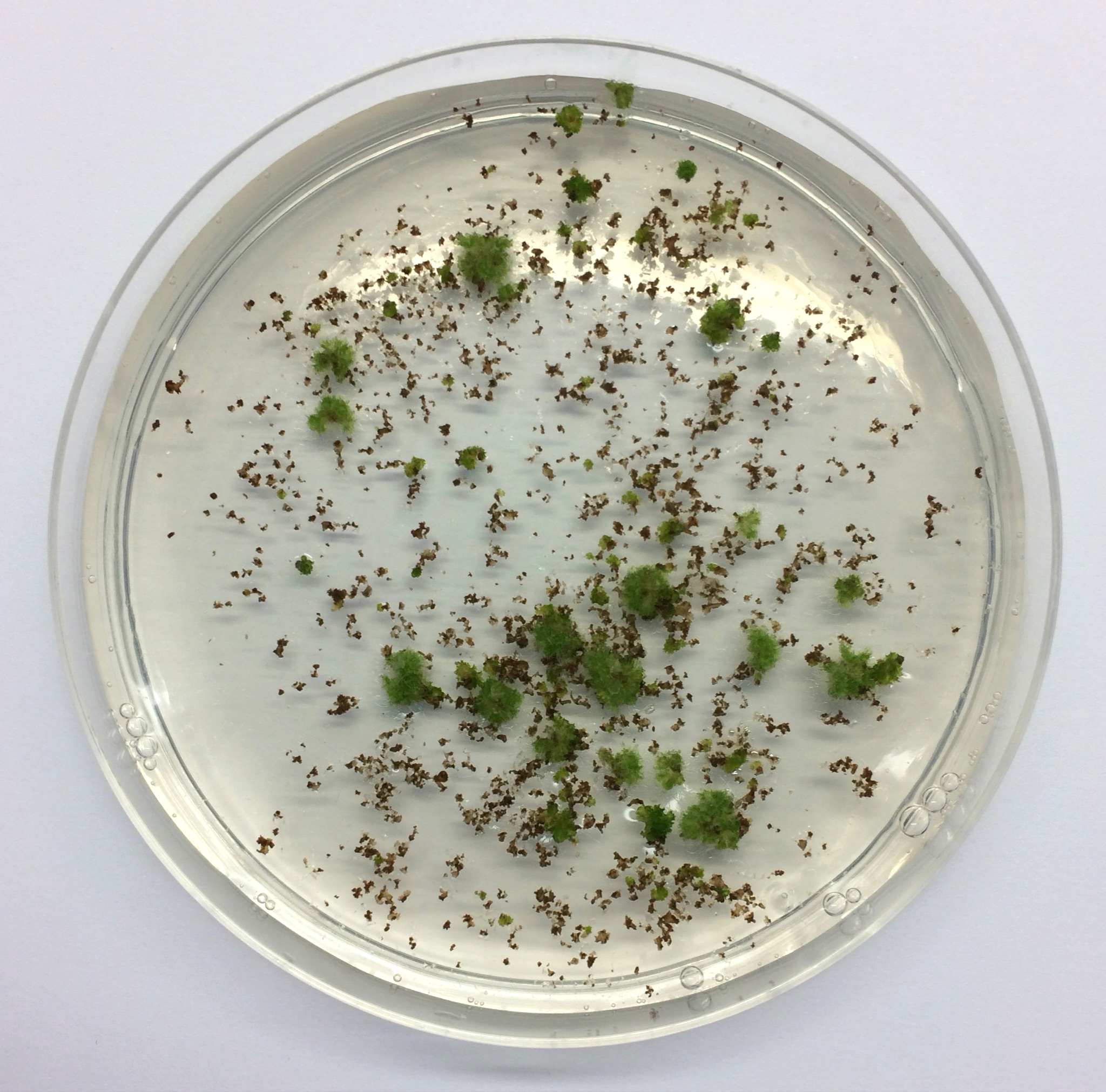
**

**Figure S7: Workflow showing steps of the protocol used to transform *A. punctatus*.**

**1)** Approximately 1 g of thallus tissue grown for 4 weeks under low light intensity was collected (approximately 0.1 g of tissue per petri dish - 10 petri dishes in total). **2)** Tissue was transferred into an empty petri dish, sterile water was added until the tissue was covered **3)** the tissue was fragmented using a razor blade (for 5 mins). **4)** the tissue was transferred from the petri dish into a cell strainer positioned on a falcon tube using a plastic pipette. **5-6)** the tissue was washed using ~100 ml of sterile water or until the flow through was clear. **7)** The fragmented thallus tissue was transferred into a 6-well plate (transfer 1⁄6 of the 1 g tissue into a single well) with 5 ml of liquid KNOP medium supplemented with 1% (w/v) sucrose and 40 mM MES, 80 μL of *Agrobacterium* culture and acetosyringone at final concentration of 100 μM. **8)** The tissue was co-cultivated with the *Agrobacterium* for 3 days on a shaker at 110 rpm, with only ambient light. **9-10)** Using a sterile plastic pipette the tissue of one well was transferred into a cell strainer, drained and then transferred on growth media containing the appropriate antibiotic (onto 1 petri dish from one well). **11)** . To facilitate spreading of the tissue, 2 ml of sterile water was added to the petri dish. **12)** After 4-6 weeks successful transformants were visible on the petri dish (successful transformants can be identified using a microscope after 4 weeks selection based on rhizoid production and/or fluorescence if such a marker is present on the construct). **13)** The emergence of rhizoids is an indication of successful transformation (yellow arrows: transformed thallus fragment, blue and pink arrows: dying thallus fragment). To eliminate false positives, surviving tissue fragments were transferred again on antibiotics containing growth media. **14)** Example of plate with successful transformants 8 weeks after co-cultivation.

Petri dish dimensions: 92 x16 mm.


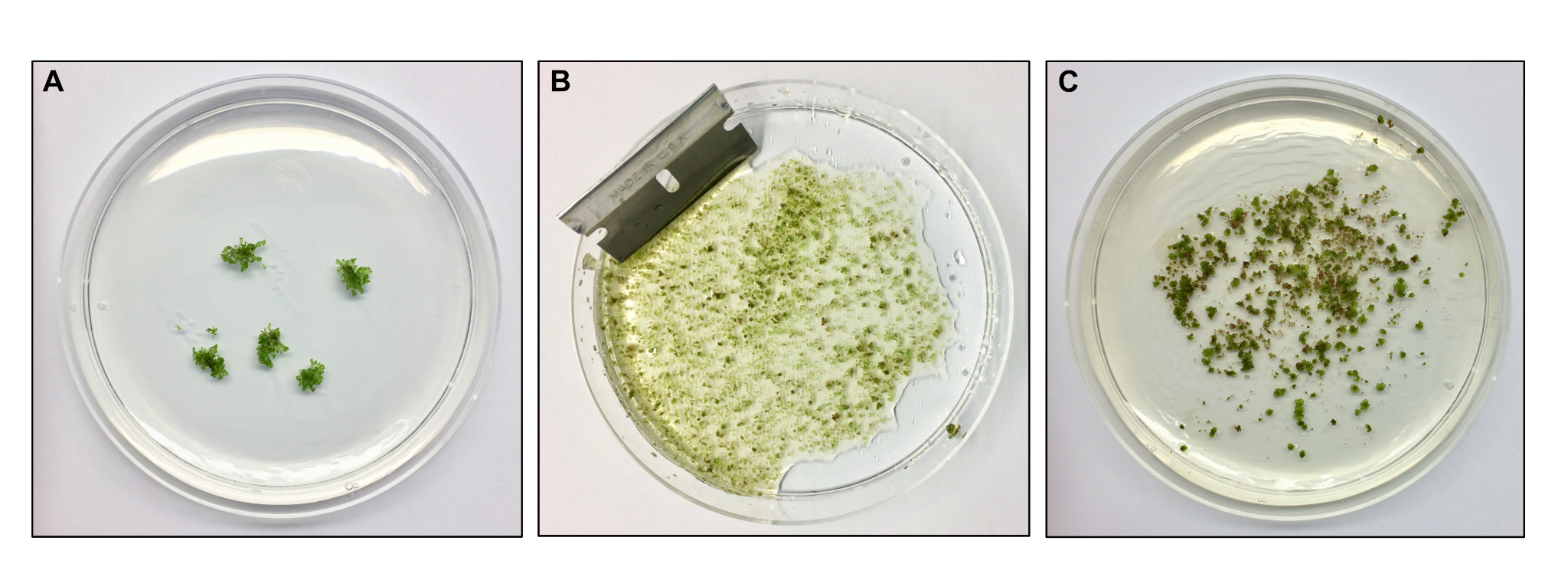


**Figure S8:** **Workflow showing steps of the protocol used to transform *L. dussii***

A) Approximately 1 g of thallus tissue grown for 4 weeks under low light intensity was collected. B) Tissue was transferred into an empty petri dish, sterile water was added until the tissue was covered and then fragmented using a razor blade (5 mins). The following steps are identical to those for the *Anthoceros* species. C) Example of plate with *L. dussii* on selection 5 weeks after co-cultivation.

Petri dish dimensions: 92 x16 mm.


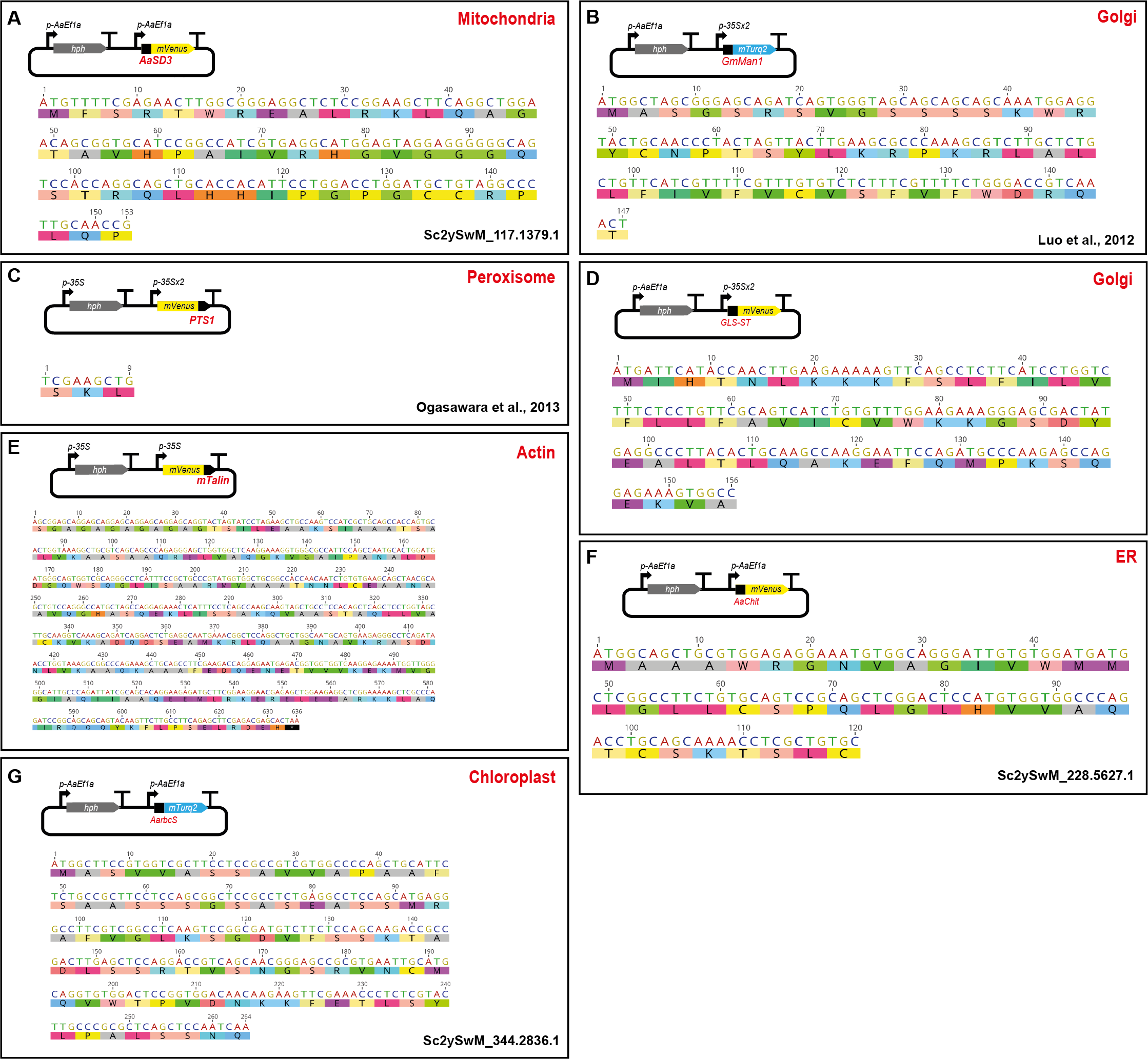


**Figure S9: Summary of sequences used as transit peptides in this study to tailor specific localization of fluorescent proteins.**

Schematic representation of the constructs, amino acid and nucleotide sequences of the targeting peptide tested for A) mitochondria (*p-AaEf1a::hph - AaEf1a::mVenus-AaSD3*), B and D) Golgi (*p-AaEf1a::hph - p-35Sx2::mTurquoise2-GmMan1*) [(Luo and Nakata 2012)](https://paperpile.com/c/sOievC/RkyO) and (*p-AaEf1a::hph - p-35Sx2::mVenus-GLS-ST*), C) Peroxisome (*p-35S::hph - p-35Sx2::mVenus-PTS1*) [(Ogasawara et al. 2013)](https://paperpile.com/c/sOievC/aLmR), E) Actin (*p-35S::hph - p-35S::mVenus-mTalin*), F) ER (*p-AaEf1a::hph - AaEf1a::mVenus-AaChit*) and G) Chloroplast (*p-AaEf1a::hph - AaEf1a::AarbcS-mTurquoise2*). All construct maps at Supp Table 1.


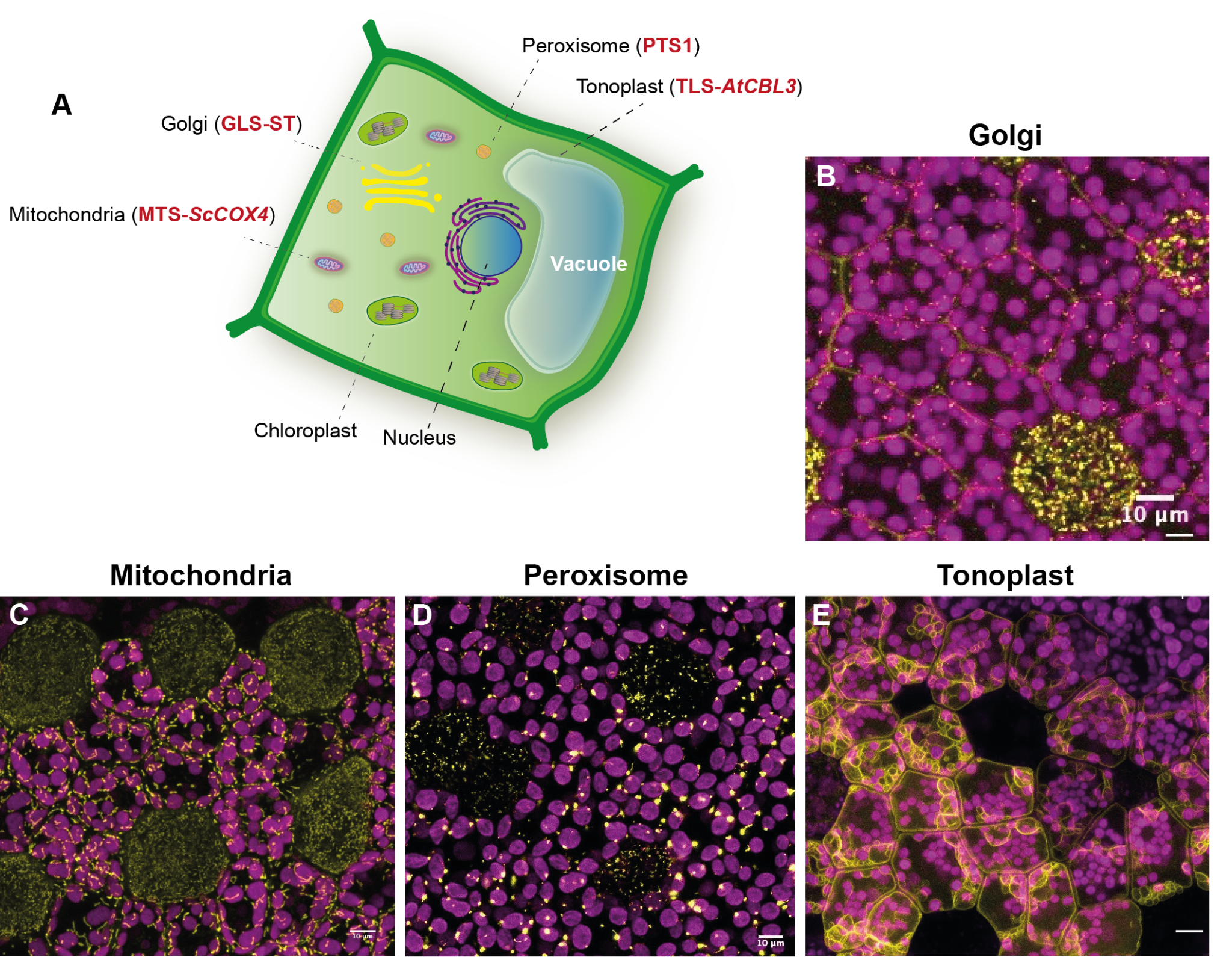


**Figure S10: Localization of fluorescent proteins tagged with various transit peptides in the liverwort *Marchantia polymorpha*.**

A) Schematic representation of a hypothetical *M. polymorpha* cell showing a summary of the subcellular localisation peptides tested in this study. B) Confocal microscopy image of *M. polymorpha* gemmae expressing the Golgi-targeted construct (*p-35S::hph - p-35Sx2::mVenus-GLS-ST*). Scale bar: 10 μm. C) Confocal microscopy image of *M. polymorpha* gemmae expressing the mitochondria-targeted construct (*p-35S::hph - p-35Sx2::mVenus-MTS-ScCOX4*). Scale bar: 10 μm. D) Confocal microscopy image of *M. polymorpha* gemmae expressing the peroxisome-targeted construct (*p-35S::hph - p-35Sx2::mVenus-PTS1*). Scale bar: 10 μm. E) Confocal microscopy image of *M. polymorpha* gemmae expressing the tonoplast-targeted construct (*p-35S::hph - p-35Sx2::mVenus-TLS-AtCBL3*). All construct maps in Supp Table 1.


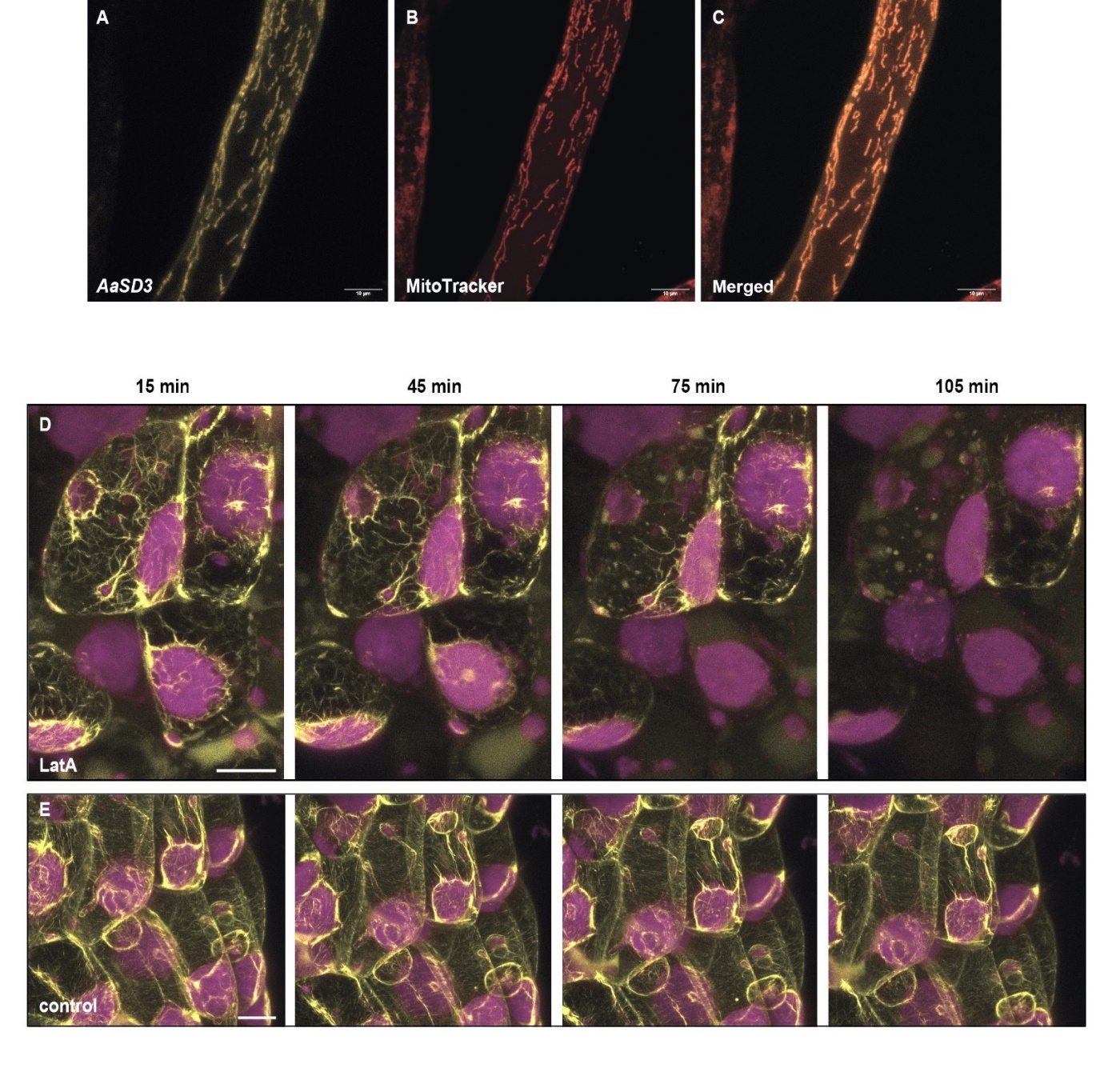


**Figure S11 MitoTracker and LatA treatment**

A) Confocal microscopy images of *A. agrestis* Oxford rhizoid, expressing the *p-AaEf1a::hph - p-AaEf1a::mVenus-AaSD3* construct. Scale bar: 10 μm. B) MitoTracker staining and C) Merged images.

D-E) Confocal microscopy images of *A. agrestis* Oxford rhizoid, expressing the *p-35S::hph - p-35S::mVenus-mTalin* construct. Actin filaments become disassembled after LatA treatment (D) but not in the control (E). Scale bars: 15 μm.


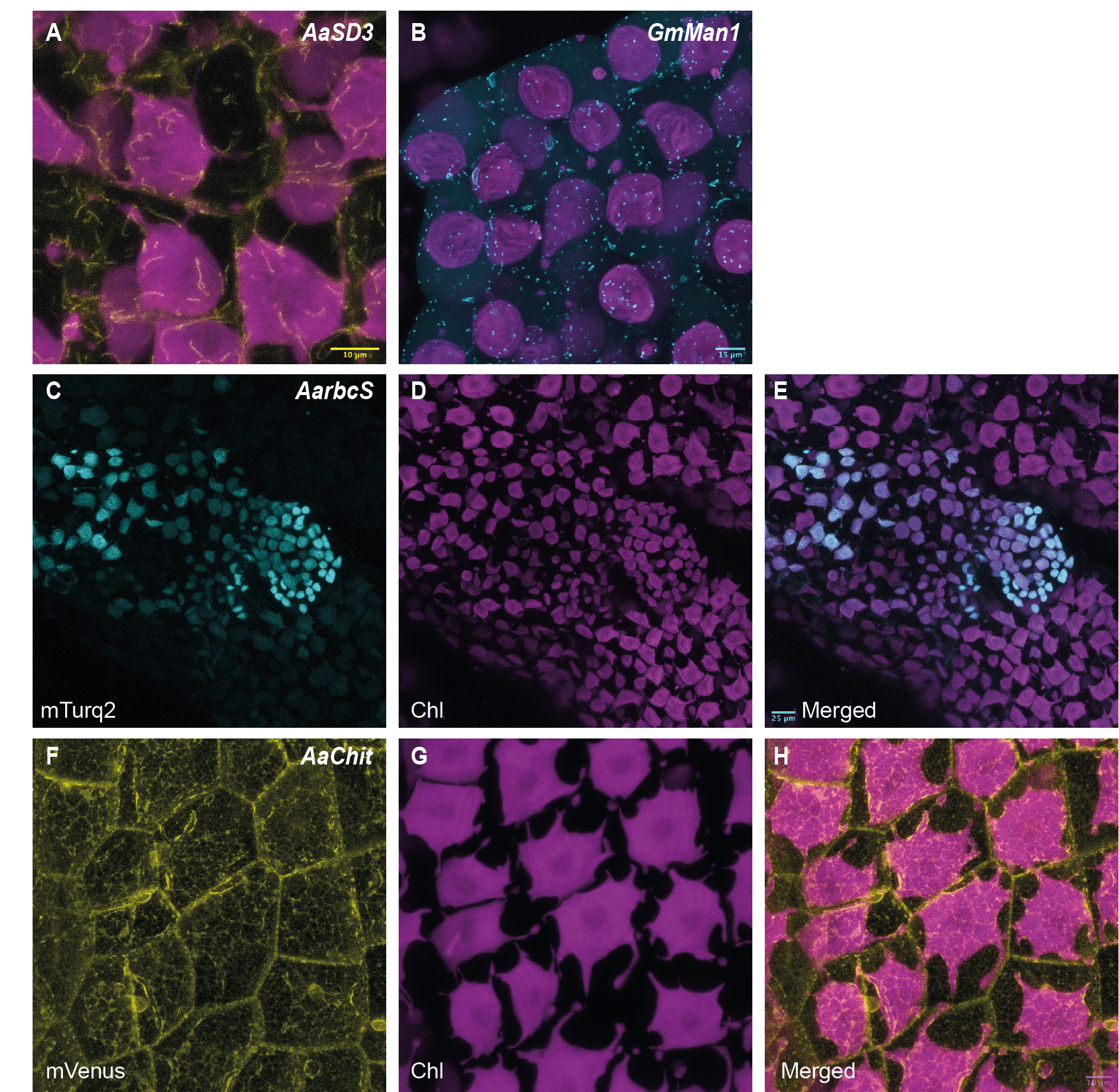


**Figure S12 Targeting fluorescent proteins to various subcellular compartments in the hornwort *A. punctatus***.

A) Confocal microscopy image of *A. punctatus* expressing the *p-AaEf1a::hph - p-AaEf1a::mVenus-AaSD3* construct. Scale bar: 10 μm B) Confocal microscopy image of *A. punctatus* expressing the *p-AaEf1a::hph - p-AaEf1a::mTurquoise-GmMan1* construct. Scale bar: 15 μm. C-E) Confocal microscopy image of *A. punctatus* expressing the *p-AaEf1a::hph - p-AaEf1a::Aa-rbcS-mTurquoise2* construct. Scale bar: 25 μm F-H) Confocal microscopy image of *A. punctatus* expressing the *p-AaEf1a::hph - p-AaEf1a::mVenus-AaChit* construct. Scale bar: 10 μm All construct maps at Supp Table 1.

**
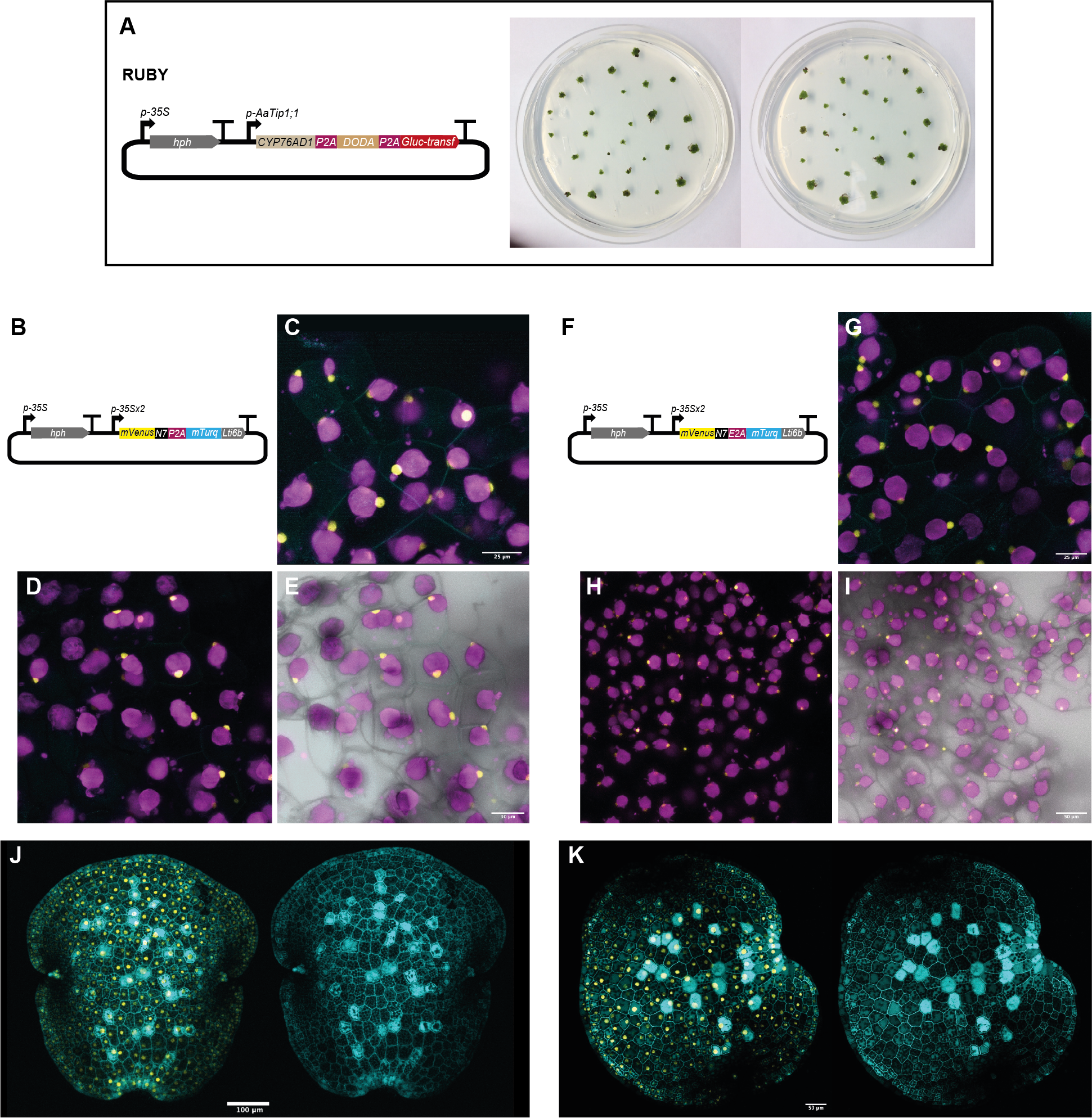
**

**Figure S13: Testing utility of the RUBY reporter and the 2A self cleavage peptides in *A. agrestis*.**

A) Right: Schematic representation of the RUBY construct. Left: Images of *A. agrestis* Oxford expressing the RUBY construct (*p-35S::hph - p-AaTip1;1::RUBY* - map Suppl Table 1). B) Schematic representation of the construct for P2A self-cleavage peptide testing (*p-35S::hph - p-35Sx2:mVenus-P2A-mTurquoise2-Lti6b*). C-E: Images of *A. agrestis* Oxford expressing the P2A self-cleavage peptide construct. J) Images of *M. polymorpha* expressing the P2A self-cleavage peptide construct. F) Schematic representation of the construct for the E2A self-cleavage peptide testing (*p-35S::hph - p-35Sx2:mVenus-N7-E2A-mTurquoise2-Lti6b*). G-I: Images of *A. agrestis* Oxford expressing the E2A self-cleavage peptide construct. K) Images of *M. polymorpha* expressing the E2A self-cleavage peptide construct. All construct maps at Supp Table 1.


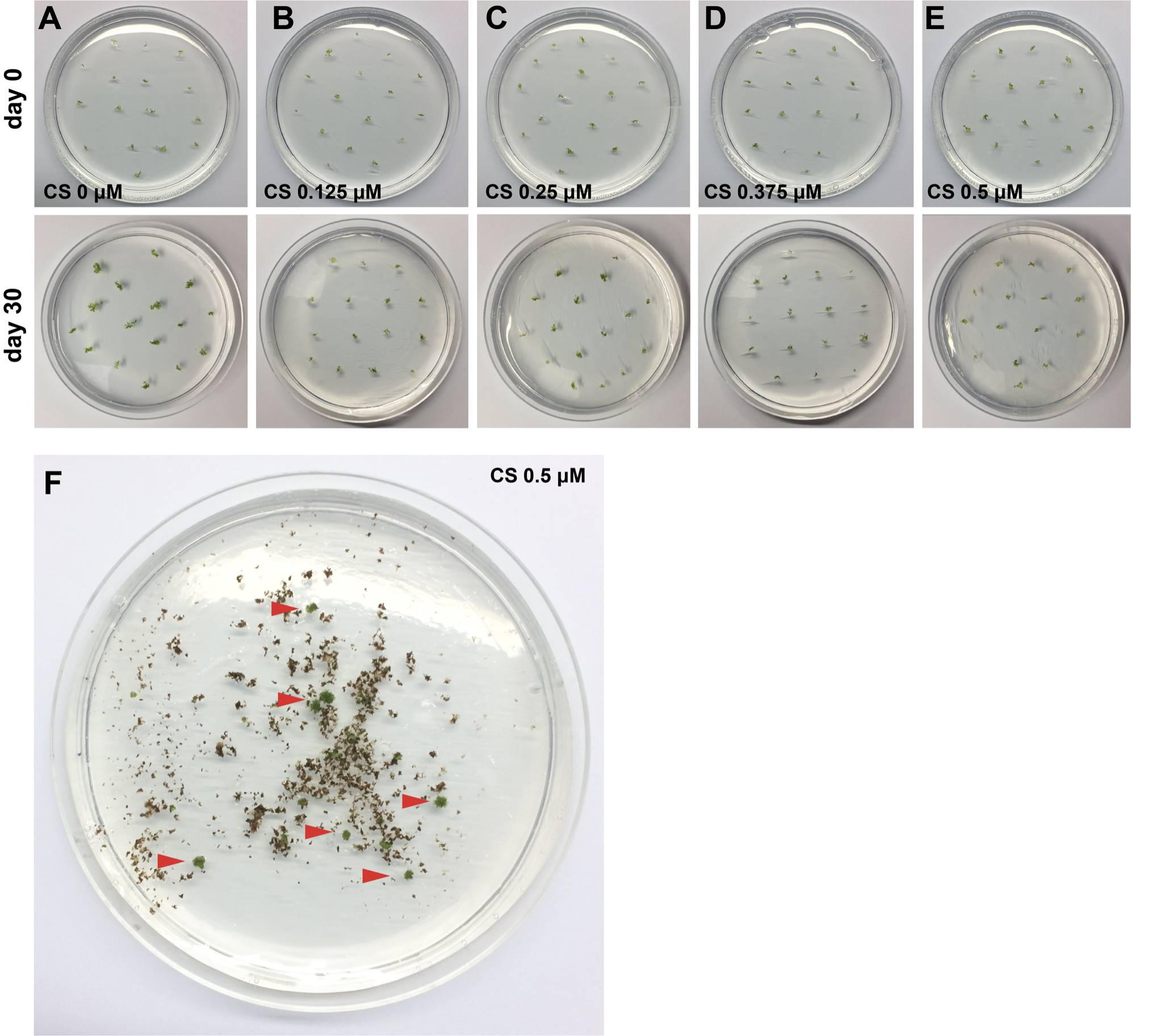


**Figure S14. Chlorsulfuron sensitivity of *A. agrestis* gametophytes.**

A-E) Plates with *A. agrestis* thallus subjected to 0 - 0.5 μM chlorsulfuron selection at day 0 of application of selection (top), day 30 (bottom). F) Example of plate with successful transformants 8 weeks after co-cultivation.

Petri dish dimensions: 92 x16 mm.

**Supplemental video 1: Supp_video1.pptx**

**Supplemental Table 1: See figshare link**

Construct sequences (genebank format, files available at 10.6084/m9.figshare.20464683):

*p-AaEf1a::hph - p-AaTip1;1::eGFP-Lti6b*

*p-AaEf1a::hph - p-AaEf1a::eGFP-Lti6b*

*p-AaEf1a::hph - p-35S_s::eGFP-Lti6b*

*p-AaEf1a::mALS - p-35S_s::eGFP-Lti6b*

*p-35S::hph - p-35Sx2:mVenus-N7-E2A-mTurquoise2-Lti6b*

*p-35S::hph - p-35Sx2:mVenus-P2A-mTurquoise2-Lti6b*

*p-AaEf1a::hph - p-35S::mScarlet-Lti6b*

*p-35S::hph - p-AaTip1;1::RUBY*

*p-AaEf1a::hph - AaEf1a::mVenus-AaSD3*

*p-AaEf1a::hph - p-35Sx2::mTurquoise2-GmMan1*

*p-35S::hph - p-35Sx2::mVenus-PTS1*

*p-35S::hph - p-35S::mVenus-mTalin*

*p-AaEf1a::hph - AaEf1a::AarbcS-mTurquoise2*

*p-AaEf1a::hph - AaEf1a::mVenus-AaChit*

*p-AaEf1a::hph - p-35Sx2::mVenus-GLS-ST*

*p-35S::hph - p-35Sx2::mVenus-TLS-AtCBL3*

*p-35S::hph - p-35Sx2::mVenus-MTS-ScCOX4*

*p-35S::hph - p-35Sx2::mVenus-GLS-ST*

**Supplemental Table 2.**

|  | *construct* | *Species* | *Number of transformants* | *Expression in rhizoids* | *Patchy expression* | *No fluorescence/ red colour* |
| --- | --- | --- | --- | --- | --- | --- |
|  | *p-35S::hph - p-35Sx2:mVenus-N7-E2A-mTurquoise2-Lti6b* | *A. agrestis*  Oxford | *16* | *2* | *14 (only mVenus)* | *1* |
|  | *p-35S::hph - p-35Sx2:mVenus-P2A-mTurquoise2-Lti6b* | *A. agrestis*  Oxford | *19* | *1* | *17 (only mVenus)* | *2* |
|  | *p-35S::hph - p-35Sx2:mVenus-N7-E2A-mTurquoise2-Lti6b* | *A. punctatus* | *7* | *-* | *7 (only mVenus)* | *-* |
|  | *p-35S::hph - p-35Sx2:mVenus-P2A-mTurquoise2-Lti6b* | *A. punctatus* | *5* | *-* | *5 (only mVenus)* | *-* |
|  | *p-35S::hph - p-AaTip1;1::RUBY* | *A. agrestis*  Oxford | *30* | *-* | *-* | *30* |

# References

Luo, B. and Nakata, P.A. (2012) ‘A set of GFP organelle marker lines for intracellular localization studies in *Medicago truncatula’*, *Plant Science*, pp. 19–24.

Ogasawara, Y. *et al.* (2013) ‘Cold-induced organelle relocation in the liverwort *Marchantia polymorpha* L’, *Plant, Cell & Environment*, pp. 1520–1528.
